# A Highly Efficient Regioselective Addition of Acetylides to Enediones Based on Steric Effects

**DOI:** 10.3390/molecules180910776

**Published:** 2013-09-03

**Authors:** Xiaoqiang Han, Chuan Wan, Dongyan Yang, Xiaoyong Yuan, Shijie Du, Yumei Xiao, Zhaohai Qin

**Affiliations:** 1 Department of Applied Chemistry, China Agricultural University, 2 West Yuanmingyuan West Road, Beijing 100193, China; E-Mails: hancau@cau.edu.cn (X.H.); wanchuan@cau.edu.cn (C.W.); yangdy@cau.edu.cn (D.Y.); gzyuanxiaoyong@163.com (X.Y.); dsj5216@163.com (S.D.); 2 Department of Chemistry, Ganzhou Teachers College, Ganzhou 341000, China

**Keywords:** regioselective addition, acetylides, enediones, steric effects, 1-ethynylcyclohex-2-enol derivatives

## Abstract

A simple and efficient strategy for the synthesis of 1-ethynylcyclohex-2-enol derivatives was developed utilizing regioselective addition of acetylides to enediones based on steric effects. Further investigation of the substrate scope of enediones indicated that all the addition reactions ocurred in good yield.

## 1. Introduction

Alkynylation reactions of carbonyl compounds that generate propargylic alcohols are among the most important carbon–carbon bond-forming reactions. Moreover, propargylic alcohols are important intermediates for the synthesis of natural products and pharmaceuticals [1,2,3]. In particular, 1-ethynyl-cyclohex-2-enol derivatives are very important intermediates used to prepare many biologically active natural products [4,5]. Recently, these derivatives have also been increasingly utilized in organic synthesis as precursors of stereodefined cyclic or acyclic molecules [6,7,8,9]. 1-Ethynylcyclohex-2-enol derivatives are easily obtained in a good yield from the reactions of cyclohexenone with alkali metal (Na, Li) alkynides or alkynyl Grignard reagents at low temperature [10,11,12]. In contrast, diketones need to be selectively protected with ethylene glycol before the alkynylation [13]. Liotta reported a useful method for preparing highly functionalized synthetic intermediates without the use of protecting groups based on stereoelectronic control [14], but the conditions of these reactions were harsh. As part of our continuing interest in the synthesis and bioactivities of 1-ethynylcyclohex-2-enol derivatives, a series of 1,4-enediones with high steric effects including substituted six-membered rings, tetralones, and quinolines were selected to react with a range of acetylides. A highly efficient regioselective addition of acetylides to 1,4-enediones without the use of a protecting group, and with only the aid of steric effects, was found. Herein, we describe this useful method and explore the 1,4-enedione and acetylide substrate scope.

## 2. Results and Discussion

4-Ketoisophorone (**1**) is a versatile substrate in the synthesis of natural products and flavors, as it has two different carbonyl groups. The 1-carbonyl group, which is connected with three methyl groups, has stronger steric hindrance than the 4-carbonyl group. Initially, using **1** as a model substrate, the regioselective addition of acetylides to **1** under various experimental conditions was investigated. As shown in Table 1, the yield of the process increased from 81% to 87% when the amount of *n*-BuLi was raised from 2.0 to 2.1 eqv (Table 1, entry 6). Decreasing the amount of THF from 30 mL to 15 mL resulted in an increase in the yield of the addition product from 82% to 87% (Table 1, entry 6). These experiments demonstrate that the best results could be obtained with 2.1 eqv of *n*-BuLi in 15 mL THF (Table 1, entry 6).

**Table 1:** Optimization of the reaction conditions ^a^.

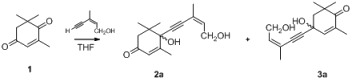				
Entry	*n*-BuLi (eqv)	Solvent (mL)	Time (h) ^b^	Yield (3a) ^c^ (%)
1	2.0	30	2	81
2	2.0	30	3	82
3	2.0	30	4	80
4	2.1	30	2	82
**5**	**2.1**	**15**	**2**	**87**
6	2.1	15	3	86
7	2.5	15	2	87

^a^ All reactions were performed on a 5-mmol scale in 4-ketoisophorone and (*Z*)-3-methylpent-2-en-4-yn-1-ol, stirred at −78 °C for 2 h and r.t. for another 2 h. ^b^ Time for reaction at r.t. ^c^ Yields for isolated products after column chromatography.

**Scheme 1:**
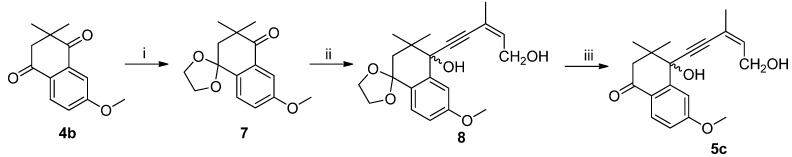
Synthesis of **5c**.

The regioselectivity of these acetylide additions can be determined using a variety of techniques, the simplest of which is proton NMR. Since the products of these additions are enones, it is quite a straightforward matter to determine if the material in question possesses an α-enone proton (high field) or a β-enone proton (low field) [14]. In **2a**, because of the dual effect of the 7′-methyl electron-donating effect and the α,β-unsaturated ketone conjugate effect, the α-H tends toward the ^1^H-NMR low field. In **3a**, the 7′-methyl electron-donating effect does not completely overlap with the α,β-unsaturated ketone conjugate effect. Thus, in the 1H-NMR, the β-H is skewed towards the high field. In ^1^H-NMR, **2a** [15] showed signals of all protons in a higher field than **3a**—*i.e.*, the α-enone proton of **2a** at 5.76 compared to the β-enone proton of **3a** at 5.85—proving the presence of steric effects (Table 2). In tetralone, in order to confirm the products’ structure, we protected the 4-carbonyl group of **4b** to give **7** [16], and after addition of acetylide and deprotection, **5c** was obtained (Scheme 1). NMR showed the presence of a significant difference (see Supplementary Material).

**Table 2:** ^1^H-NMR of **2a** and **3a**.

	2a ^a^	3a ^b^
=CH	5.83	5.92
=CH	5.76	5.85
CH_2_OH	4.17	4.25
CH_2_	2.37	2.46
vinyl CH_3_	2.05	2.14
vinyl CH_3_	1.79	1.87
CH_3_	1.12	1.23
CH_3_	1.03	1.11
CH_2_OH	3.3	3.49

^a^ 360 MHz, using CDCl_3_ as the solvent. ^b^ 300 MHz, using CDCl_3_ as the solvent.

*Conditions and reagents*: i. *p*-TSA, CH(OCH_3_)_3_, HOCH_2_CH_2_OH, ether; ii. *n*-BuLi, (*z*)-3-methylpent-2-en-4-yn-1-ol, THF, −78 °C; iii. *p*-TSA, acetone.

With the optimal conditions identified, the scope of the regioselective additions of acetylides to enediones based on steric effects was investigated (Table 3 and Table 4). The results of these processes, summarized in Table 3 and Table 4, show that these processes take place efficiently to generate adducts in good to excellent yields. With additions of acetylides to 4-ketoisophorone, the propionates, *t*-butylacetylene, and phenylethyne, gave excellent yields (Table 3, entries 3–6), but alkynols gave lower yields (Table 3, entry 1). The best yield was obtained when ethyl propiolate was used as a substrate (Table 3, entry 3).

**Table 3:** Regioselective additions of acetylides to 4-ketoisophorone.

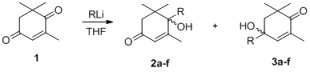				
Entry	Substrate	RLi	2 (%)	3 (%) ^a^
1	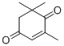	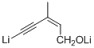	—	3a: 87
2	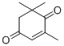	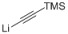	—	3b: 91
3	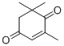	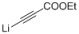	—	3c: 95
4	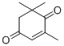	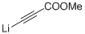	—	3d: 92
5	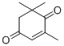	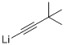	—	3e: 93
6	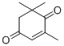	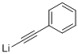	—	3f: 94

^a^ Yields for isolated products after column chromatography.

Further investigations demonstrated that substituent effects influence the yields of tertiary propargylic alcohol formation in the regioselective additions of acetylides to dimethyl dihydronaphthalene-1,4-diones. The data in Table 4 show that dimethyl dihydronaphthalene-1,4-diones with electron-withdrawing or -donating substituents at the *meta* and *para* positions of the benzene rings worked well (Table 4, entries 3–8). It is worth mentioning that dimethyl dihydroquinoline-5,8-diones also worked well, readily affording the corresponding products with the best yield and regioselectivity (Table 4, entries 9–11). Similarly, a significant yield was obtained when ethyl propiolate was used as a substrate (Table 4, entries 2 and 6).

**Table 4:** Regioselective additions of acetylides to dimethyl dihydronaphthalene-1,4-diones.

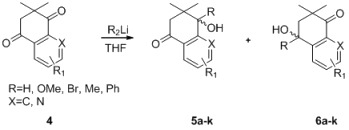				
Entry	R_1_, X [17,18]	R_2_Li	5 (%)	6 (%) ^a^
1	4a_:_ R_1_ = H, X = C	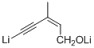	—	**6a**: 87
2	4a_:_ R_1_ = H, X = C	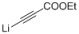	—	**6b**: 94
3	4b_:_ R_1_ = 7-Ome, X = C	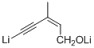	—	**6c**: 86
4	4c_:_ R_1_ = 6-Ome, X = C	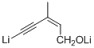	—	**6d**: 84
5	4c_:_ R_1_ = 6-Ome, X = C	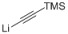	—	**6e**: 88
6	4c_:_ R_1_ = 6-Ome, X = C	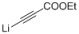	—	**6f**: 94
7	4c_:_ R_1_ = 6-Ome, X = C	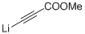	—	**6g**: 91
8	4d_:_ R_1_ = 7-Br, X = C	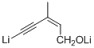	—	**6h**: 89
9	4e_:_ R_1_ = 2-Me, X = N	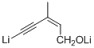	—	**6i**: 94
10	4f_:_ R_1_ = 2-Ph, X = N	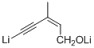	—	**6j**: 96
11	4g_:_ R_1_ = H, X = N	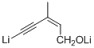	—	**6k**:95

^a^ Yields for isolated products after column chromatography.

## 3. Experimental

The ^1^H and ^13^C-NMR spectra were recorded on a Bruker Avance DPX300 with tetramethylsilane as an internal standard, all NMR spectra were obtained using CDCl_3_ as the solvent unless otherwise noted. GC-Mass was carried out on a 6890N GC-Agilent 5973N. Melting points were measured on an Cole-Parmer melting point apparatus and are not corrected. Commercially available compounds were used in this work without further purification. The solvent tetrahydrofuran (THF) was dried by distillation from sodium and benzophenone. Substrates **4** were synthesized by our lab, while the others were commercially available. Column chromatography was performed using silica gel (200–300 mesh). TLC was performed on GF254 silica gel plates (Qingdao Haiyang Co., Ltd, Qingdao, China). NMR spectra were recorded on a Bruker Avance DPX300 spectrometer (300 MHz for ^1^H and 75 MHz for ^13^C) with tetramethylsilane as the internal standard.

*(Z)-4-Hydroxy-4-(5′-hydroxy-3′-methylpent-3′-en-1′-yn-1′-yl)-2,6,6-trimethylcyclohex-2-enone* (**3a**): *n*-BuLi (4.2 mL, 10 mmol, 2.4 mol/L) was added dropwise to a solution of (*Z*)-3-methylpent-2-en-4-yn-1-ol (0.48 g, 5 mmol) in THF (10 mL) at −78 °C under a N_2_ atmosphere, and then the mixture was stirred for 1 h at −78 °C. A solution of 4-ketoisophorone **1** (0.76 g, 5 mmol) in THF (5 mL) was added slowly, the mixture was stirred for 0.5 h at −78 °C, and then it was warmed to room temperature and stirred for 2 h. The reaction was quenched with saturated aqueous NH_4_Cl solution (10 mL) and extracted with EtOAc (3 × 30 mL). The combined organics were washed with brine (2 × 20 mL), dried over anhydrous Na_2_SO_4_ and concentrated. The residue was purified by silica gel column chromatography (petroleum ether/EtOAc, 2:1) to afford **3a** (1.08 g, 87%) as a red-brown oil. ^1^H-NMR (CDCl_3_, 25 °C): δ 5.94–5.91 (m, 1H), 5.90–5.84 (m, 1H), 4.41 (s, 1H), 4.26–4.24 (d, *J* = 6.7, 2H), 3.49 (s, 1H), 2.53–2.39 (m, 2H), 2.14 (s, 3H), 1.87 (s, 3H), 1.23 (s, 3H), 1.11 (s, 3H). ^13^C-NMR (CDCl_3_, 25 °C) : δ 199.0, 136.7, 125.5, 119.8, 92.7, 85.1, 74.4, 60.6, 41.7, 25.0, 22.7, 19.7.

*4-Hydroxy-2,6,6-trimethyl-4-((trimethylsilyl)ethynyl)cyclohex-2-enone* (**3b**): *n*-BuLi (2.1 mL, 5 mmol, 2.4 mol/L) was added dropwise to a solution of ethynyltrimethylsilane (0.49 g, 5 mmol) in THF (10 mL) at −78 °C under a N_2_ atmosphere, and then the mixture was stirred for 1 h at −78 °C. A solution of 4-ketoisophorone **1** (0.76 g, 5 mmol) in THF (5 mL) was added slowly, the mixture was stirred for 0.5 h at −78 °C, and then it was warmed to room temperature and stirred for 2 h. The reaction was quenched with saturated aqueous NH_4_Cl solution (10 mL) and extracted with EtOAc (3 × 30 mL). The combined organics were washed with brine (2 × 20 mL), dried over anhydrous Na_2_SO_4_ and concentrated. The residue was purified by silica gel column chromatography (petroleum ether/EtOAc, 5:1) to afford **3b** (1.14 g, 91%) as a white solid, mp: 76–78 °C. ^1^H-NMR (CDCl_3_, 25 °C) δ 5.77 (d, *J* = 1.1 Hz, 1H), 2.71 (s, 1H), 2.38 (q, *J*_1_ = 16.4 Hz, *J*_2_ = 37.2 Hz, 2H), 2.04 (d, J = 1.3 Hz, 3H), 1.12 (s, 3H), 1.06 (s, 3H), 0.10 (s, 9H). ^13^C-NMR (CDCl_3_, 25 °C) δ 198.4, 126.0, 103.9, 92.2, 74.6, 58.2, 49.0, 41.5, 24.9, 19.5, 18.2, -0.3.MS:273.1 (M + Na^+^).

*Ethyl 3-(1′-hydroxy-3′,5′,5′-trimethyl-4′-oxocyclohex-2′-en-1′-yl)propiolate* (**3c**): LDA (2.5 mL, 5 mmol, 2.0 mol/L) was added dropwise to a solution of ethylpropiolate (0.49 g, 5 mmol) in THF (10 mL) at −78 °C under a N_2_ atmosphere, and then the mixture was stirred for 1 h at −78 °C. A solution of 4-ketoisophorone **1** (0.76 g, 5 mmol) in THF (5 mL) was added slowly, the mixture was stirred for 0.5 h at −78 °C, and then it was warmed to room temperature and stirred for 2 h. The reaction was quenched with saturated aqueous NH_4_Cl solution (10 mL) and extracted with EtOAc (3 × 30 mL). The combined organics were washed with brine (2 × 20 mL), dried over anhydrous Na_2_SO_4_ and concentrated. The residue was purified by silica gel column chromatography (petroleum ether/EtOAc, 10:1) to afford **3c** (1.19 g, 95%) as a white solid, 72–73 °C. ^1^H-NMR (CDCl_3_, 25 °C) δ 5.91 (d, *J* = 1.3 Hz, 1H), 4.25 (q, *J*_1_ = 7.1 Hz, *J*_2_ = 14.2 Hz, 2H), 3.52 (s, 1H), 2.47 (t, *J* = 12.0 Hz, 2H), 2.14 (d, *J* = 1.4 Hz, 3H), 1.32 (t, *J* = 7.2 Hz, 3H), 1.24 (s, 3H), 1.12 (s, 3H). ^13^C-NMR (CDCl_3_, 25 °C) δ. 197.7, 158.2, 152.8, 126.8, 85.0, 78.4, 74.4, 62.3, 48.7, 42.0, 24.9, 21.7, 19.6, 13.8. MS:273.0 (M + Na^+^).

*Methyl 3-(1′-hydroxy-3′,5′,5′-trimethyl-4′-oxocyclohex-2′-en-1′-yl)propiolate* (**3d**): LDA (2.5 mL, 5 mmol, 2.0 mol/L) was added dropwise to a solution of methyl propiolate (0.42 g, 5 mmol) in THF (10 mL) at −78 °C under a N_2_ atmosphere, and then the mixture was stirred for 1 h at −78 °C. A solution of 4-ketoisophorone **1** (0.76 g, 5 mmol) in THF (5 mL) was added slowly, the mixture was stirred for 0.5 h at −78 °C, and then it was warmed to room temperature and stirred for 2 h. The reaction was quenched with saturated aqueous NH_4_Cl solution (10 mL) and extracted with EtOAc (3 × 30 mL). The combined organics were washed with brine (2 × 20 mL), dried over anhydrous Na_2_SO_4_ and concentrated. The residue was purified by silica gel column chromatography (petroleum ether/EtOAc, 9:1) to afford **3d** (1.08 g, 92%) as a red-brown oil. ^1^H-NMR (CDCl_3_, 25 °C) δ 5.92 (d, *J* = 1.3 Hz, 1H), 4.22 (s, 1H), 3.79 (s, 3H), 2.49 (q, *J* = 6.6 Hz, 2H), 2.15 (d, *J* = 1.3 Hz, 3H), 1.25 (s, 3H), 1.13 (s, 3H). ^13^C-NMR (CDCl_3_, 25 °C) δ 198.0, 153.2, 126.6, 85.7, 77.4, 74.2, 52.8, 41.9, 24.8, 19.5. MS:258.9 (M + Na^+^).

*4-(3′,3′-Dimethylbut-1′-yn-1′-yl)-4-hydroxy-2,6,6-trimethylcyclohex-2-enone* (**3e**): *n*-BuLi (2.1 mL, 5 mmol, 2.4 mol/L) was added dropwise to a solution of 3,3-dimethyl-1-butyne (0.41 g, 5 mmol) in THF (10 mL) at −78 °C under a N_2_ atmosphere, and then the mixture was stirred for 1 h at –78 °C. A solution of 4-ketoisophorone **1** (0.76 g, 5 mmol) in THF (5 mL) was added slowly, the mixture was stirred for 0.5 h at −78 °C, and then it was warmed to room temperature and stirred for 2 h. The reaction was quenched with saturated aqueous NH_4_Cl solution (10 mL) and extracted with EtOAc (3 × 30 mL). The combined organics were washed with brine (2 × 20 mL), dried over anhydrous Na_2_SO_4_ and concentrated. The residue was purified by silica gel column chromatography (petroleum ether /EtOAc, 10:1) to afford **3e** (1.09 g, 93%) as a red-brown oil. ^1^H-NMR (CDCl_3_, 25 °C) δ 5.81 (d, *J* = 1.0 Hz, 1H), 3.0 (s, 1H), 2.43 (d, *J* = 8.5 Hz, 2H), 2.11 (d, *J* = 1.2 Hz, 3H), 1.22 (s, 9H), 1.17 (s, 3H), 1.09 (s, 3H). ^13^C-NMR (CDCl_3_, 25 °C) δ 198.6, 125.4, 95.9, 74.0, 41.5, 30.6, 27.4, 24.9, 19.6. MS:257.0 (M + Na^+^).

*4-Hydroxy-2,6,6-trimethyl-4-(phenylethynyl)cyclohex-2-enone* (**3f**): *n*-BuLi (2.1 mL, 5 mmol, 2.4 mol/L) was added dropwise to a solution of phenylacetylene (0.51 g, 5 mmol) in THF (10 mL) at −78 °C under a N_2_ atmosphere, and then the mixture was stirred for 1 h at −78 °C. A solution of 4-ketoisophorone **1** (0.76 g, 5 mmol) in THF (5 mL) was added slowly, the mixture was stirred for 0.5 h at −78 °C, and then it was warmed to room temperature and stirred for 2 h. The reaction was quenched with saturated aqueous NH_4_Cl solution (10 mL) and extracted with EtOAc (3 × 30 mL). The combined organics were washed with brine (2 × 20 mL), dried over anhydrous Na_2_SO_4_ and concentrated. The residue was purified by silica gel column chromatography (petroleum ether/EtOAc, 10:1) to afford **3f** (1.19 g, 94%) as a red-brown oil. ^1^H-NMR (CDCl_3_, 25 °C) δ 7.42–7.38 (m, 2H), 7.32–7.26 (m, 3H), 5.89 (d, *J* = 1.3 Hz, 1H), 3.88 (s, 1H), 2.50 (q, *J*_1_ = 19.2 Hz, *J*_2_ = 23.2 Hz, 2H), 2.16 (d, *J* = 1.4 Hz, 3H), 1.22 (s, 3H), 1.14 (s, 3H). ^13^C-NMR (CDCl_3_, 25 °C) δ 198.7, 131.5, 128.6, 128.1, 125.6, 121.8, 87.7, 86.7, 76.5, 74.4, 49.1, 41.8, 24.9, 21.7, 19.6. MS:277.0 (M + Na^+^).

*(Z)-4-hydroxy-4-(5′-hydroxy-3′-methylpent-3′-en-1′-yn-1′-yl)-6-methoxy-3,3-dimethyl-3,4-dihydronaphthalen-1(2H)-one* (**5c**): To a stirred solution of **4b** (2.0 g, 9.2 mmol), ethylene glycol (1.14 g, 18.4 mmol), methylorthoformate (1.46 g, 13.8 mmol) and *p*-TSA (0.15g, 0.87 mmol) in 150 mL diethyl ether under an atmosphere of argon at r.t for 16h. The reaction was quenched by slow addition of aqueous NaHCO_3_ solution (5 mL) and extracted with diethyl ether (3 × 50 mL). The organic phase was washed with water (2 × 50 mL) and dried over anhydrous Na_2_SO_4_ and filtered, filtrate concentrated under reduced pressure. The residue was subjected to silica gel chromatography using PE and EtOAc (6:1) as eluant to afford ketal **7** as a yellow oil (2.05 g, yield 85%). **7**: ^1^H-NMR (CDCl_3_, 25 °C) δ 7.49–7.46 (m, 2H), 7.14 (dd, *J*_1_ = 2.8 Hz, *J*_2_ = 8.5 Hz, 1H), 4.22–4.08 (m, 4H), 3.84 (s, 3H), 2.20 (s, 2H), 1.30 (s, 6H). ^13^C-NMR (CDCl_3_, 25 °C) δ 201.9, 160.2, 135.2, 132.3, 127.3, 121.5, 109.2, 105.0, 64.9, 55.3, 45.3, 42.4, 26.6.

*n*-BuLi (4.2 mL, 10 mmol, 2.4 mol/L) was added dropwise to a solution of (*Z*)-3-methylpent-2-en-4-yn-1-ol (0.48 g, 5 mmol) in THF (10 mL) at −78 °C under a N_2_ atmosphere, and then the mixture was stirred for 1 h at −78 °C. A solution of **7** (1.31 g, 5 mmol) in THF (5 mL) was added slowly, the mixture was stirred for 0.5 h at −78 °C, and then it was warmed to room temperature and stirred for 2 h. The reaction was quenched with saturated aqueous NH_4_Cl solution (10 mL) and extracted with EtOAc (3 × 30 mL). The combined organics were washed with brine (2 × 20 mL), dried over anhydrous Na_2_SO_4_ and concentrated. The residue was purified by silica gel column chromatography (petroleum ether/EtOAc, 2:1) to afford **8** (1.54 g, 86%) as a yellow oil. **8**: ^1^H-NMR (300 MHz, CDCl_3_, 25 °C) δ 7.39 (d, *J* = 7.5 Hz, 1H), 7.31 (d, *J* = 2.6 Hz, 1H), 6.89 (dd, *J*_1_ = 2.6 Hz, *J*_2_ = 8.6 Hz, 1H), 5.85 (t, *J* = 1.4 Hz, 1H), 4.22 (d, *J* = 6.8 Hz, 2H), 4.17-4.05 (m, 4H), 3.81 (s, 3H), 3.08 (s, 1H), 2.22 (d, *J* = 14.2 Hz, 1H), 2.03 (d, *J* = 14.2 Hz, 1H), 1.87 (d, *J* = 0.9 Hz, 3H), 1.15 (s, 3H), 1.12 (s, 3H). ^13^C-NMR (75 MHz, CDCl_3_, 25 °C) δ 160.1, 141.4, 136.1, 128.7, 127.6, 120.3, 120.3, 114.9, 111.0, 105.8, 95.2, 85.0, 75.1, 64.6, 60.9, 55.2, 42.9, 39.2, 25.2, 23.5, 22.9.

To a stirred solution of 8 (1.50 g, 4.2 mmol) in acetone (30 mL), *p*-TSA (60 mg) was added, and the mixture was stirred for 1 h at room temperature. A saturated NaHCO_3_ solution (5 mL) was added, and it was extracted with EtOAc (3 × 30 mL). The combined organics were washed with brine (3 × 20 mL), dried over anhydrous Na_2_SO_4_ and concentrated to a residue, which was purified by silica gel column chromatography (petroleum ether/EtOAc, 2:1) to afford **5c** (1.17 g, 89%) as a yellow oil. ^1^H-NMR (CDCl_3_, 25 °C) δ 7.99 (d, *J* = 8.6 Hz, 1H), 7.39 (d, *J* = 2.5 Hz, 1H), 6.92 (dd, *J*_1_ = 2.5 Hz, *J*_2_ = 8.7 Hz, 1H), 5.94-5.89 (m, 1H), 4.29 (d, *J* = 6.7 Hz, 2H), 3.90 (s, 3H), 2.86-2.63 (m, 3H), 1.90 (s, 3H), 1.17 (s, 6H). ^13^C-NMR (CDCl_3_, 25 °C) δ 195.7, 164.4, 136.6, 130.8, 129.5, 128.8, 123.6, 120.0, 114.2, 94.2, 85.9, 74.8, 65.5, 61.2, 55.5, 41.5, 30.5, 24.9, 22.9.

*(Z)-4-Hydroxy-4-(5′-hydroxy-3′-methylpent-3′-en-1′-yn-1′-yl)-2,2-dimethyl-3,4-dihydronaphthalen-1(2H)-one* (**6a**): *n*-BuLi (4.2 mL, 10 mmol, 2.4 mol/L) was added dropwise to a solution of (*Z*)-3-methylpent-2-en-4-yn-1-ol (0.48 g, 5 mmol) in THF (10 mL) at −78 °C under a N_2_ atmosphere, and then the mixture was stirred for 1 h at −78 °C. A solution of **4a** (0.94 g, 5 mmol) in THF (5 mL) was added slowly, the mixture was stirred for 0.5 h at –78 °C, and then it was warmed to room temperature and stirred for 2 h. The reaction was quenched with saturated aqueous NH_4_Cl solution (10 mL) and extracted with EtOAc (3 × 30 mL). The combined organics were washed with brine (2 × 20 mL), dried over anhydrous Na_2_SO_4_ and concentrated. The residue was purified by silica gel column chromatography (petroleum ether/EtOAc, 2:1) to afford **6a** (1.24 g, 87%) as a yellow oil. ^1^H-NMR (CDCl_3_, 25 °C) δ 8.02 (d, *J* = 9.2 Hz, 1H), 7.90 (d, *J* = 8.9 Hz, 1H), 7.63 (t, *J* = 7.4 Hz, 1H), 7.44 (t, *J* = 7.6 Hz, 1H), 5.94–5.89 (m, 1H), 4.28 (d, *J* = 6.6 Hz, 2H), 2.94 (d, *J* = 17.4 Hz, 2H), 2.64 (d, *J* = 17.4 Hz, 2H), 1.91 (d, *J* = 1.1 Hz, 3H), 1.77 (s, 2H), 1.21 (s, 3H), 1.17 (s, 3H). ^13^C-NMR (CDCl_3_, 25 °C) δ 197.1, 144.0, 136.5, 134.3, 130.0, 128.6, 127.0, 126.9, 120.0, 94.3, 85.9, 74.7, 61.25, 48.5, 41.4, 25.0, 23.1, 22.9.

*Ethyl 3-(1′-hydroxy-3′,3′-dimethyl-4′-oxo-1′,2′,3′,4′-tetrahydronaphthalen-1′-yl)propiolate* **6b**: LDA (2.5 mL, 5 mmol, 2.0 mol/L) was added dropwise to a solution of ethylpropiolate (0.49 g, 5 mmol) in THF (10 mL) at −78 °C under a N_2_ atmosphere, and then the mixture was stirred for 1 h at −78 °C. A solution of **4a** (0.94 g, 5 mmol) in THF (5 mL) was added slowly, the mixture was stirred for 0.5 h at −78 °C, and then it was warmed to room temperature and stirred for 2 h. The reaction was quenched with saturated aqueous NH_4_Cl solution (10 mL) and extracted with EtOAc (3 × 30 mL). The combined organics were washed with brine (2 × 20 mL), dried over anhydrous Na_2_SO_4_ and concentrated. The residue was purified by silica gel column chromatography (petroleum ether/EtOAc, 10:1) to afford **6b** (1.34 g, 94%) as a red-brown liquid. ^1^H-NMR (CDCl_3_, 25 °C) δ 8.00 (dd, *J*_1_ = 1.0 Hz, *J*_2_ = 7.7 Hz, 1H), 7.89 (d, *J* = 7.5 Hz, 1H), 7.62 (dt, *J*_1_ = 1.3 Hz, *J*_2_ = 7.5 Hz, 1H), 4.22 (q, *J*_1_ = 7.1 Hz, *J*_2_ = 14.2 Hz, 2H), 3.97 (s, 1H), 2.90 (d, *J* = 17.3 Hz, 1H), 2.63 (d, *J* = 17.3 Hz, 1H), 1.29 (t, *J* = 7.1 Hz, 3H), 1.22 (s, 3H), 1.18 (s, 3H). ^13^C-NMR (CDCl_3_, 25 °C) δ 196.7, 153.0, 142.2, 134.4, 129.8, 129.0, 127.3, 126.9, 86.4, 79.0, 74.1, 62.2, 41.4, 24.6, 13.8. MS:309.0 (M+Na^+^).

*(Z)-4-Hydroxy-4-(5′-hydroxy-3′-methylpent-3′-en-1′-yn-1′-yl)-7-methoxy-2,2-dimethyl-3,4-dihydronaphthalen-1(2H)-one* (**6c**): *n*-BuLi (4.2 mL, 10 mmol, 2.4 mol/L) was added dropwise to a solution of (*Z*)-3-methylpent-2-en-4-yn-1-ol (0.48 g, 5 mmol) in THF (10 mL) at –78 °C under a N_2_ atmosphere, and then the mixture was stirred for 1 h at –78 °C. A solution of **4b** (1.09 g, 5 mmol) in THF (5 mL) was added slowly, the mixture was stirred for 0.5 h at −78 °C, and then it was warmed to room temperature and stirred for 2 h. The reaction was quenched with saturated aqueous NH_4_Cl solution (10 mL) and extracted with EtOAc (3 × 30 mL). The combined organics were washed with brine (2 × 20 mL), dried over anhydrous Na_2_SO_4_ and concentrated. The residue was purified by silica gel column chromatography (petroleum ether/EtOAc, 2:1) to afford **6c** (1.35 g, 86%) as a yellow oil. ^1^H-NMR (CDCl_3_, 25 °C) δ 7.96 (d, *J* = 8.7 Hz, 1H), 7.39 (d, *J* = 2.4 Hz, 1H), 6.89 (dd, *J*_1_ = 2.4 Hz, *J*_2_ = 8.7 Hz, 1H), 5.91–5.87 (m, 1H), 4.26 (d, *J* = 6.4 Hz, 2H), 3.87 (s, 3H), 2.78-2.61 (m, 3H), 1.87 (s, 3H), 1.15 (s, 6H). ^13^C-NMR (CDCl_3_, 25 °C) δ 196.2, 164.3, 146.8, 136.4, 129.3, 123.4, 119.9, 114.0, 111.8, 94.1, 85.6, 74.4, 60.8, 55.4, 48.2, 41.4, 30.7, 24.8, 22.8.

*(Z)-4-Hydroxy-4-(5′-hydroxy-3′-methylpent-3′-en-1′-yn-1′-yl)-6-methoxy-2,2-dimethyl-3,4-dihydronaphthalen-1(2H)-one* (**6d**): *n*-BuLi (4.2 mL, 10 mmol, 2.4 mol/L) was added dropwise to a solution of (*Z*)-3-methylpent-2-en-4-yn-1-ol (0.48 g, 5 mmol) in THF (10 mL) at −78 °C under a N_2_ atmosphere, and then the mixture was stirred for 1 h at –78 °C. A solution of **4c** (1.09 g, 5 mmol) in THF (5 mL) was added slowly, the mixture was stirred for 0.5 h at –78 °C, and then it was warmed to room temperature and stirred for 2 h. The reaction was quenched with saturated aqueous NH_4_Cl solution (10 mL) and extracted with EtOAc (3 × 30 mL). The combined organics were washed with brine (2 × 20 mL), dried over anhydrous Na_2_SO_4_ and concentrated. The residue was purified by silica gel column chromatography (petroleum ether /EtOAc, 2:1) to afford **6d** (1.32 g, 84%) as a yellow oil. ^1^H-NMR (CDCl_3_, 25 °C) δ 7.81 (d, *J* = 8.6 Hz, 1H), 7.46 (d, *J* = 2.8 Hz, 1H), 7.13 (dd, *J*_1_ = 2.8 Hz, *J*_2_ = 8.6 Hz, 1H), 5.90–5.85 (m, 1H), 4.27 (d, *J* = 6.7 Hz, 2H), 3.83 (s, 3H), 2.88 (d, *J* = 16.8 Hz, 1H), 2.58 (d, *J* = 16.8 Hz, 1H), 1.88 (d, *J* = 0.78 Hz, 3H), 1.20 (s, 3H), 1.13 (s, 3H). ^13^C-NMR (CDCl_3_, 25 °C) δ 197.4, 159.5, 136.7, 136.2, 131.0, 128.9, 121.5, 120.0, 109.3, 94.4, 85.4, 74.1, 60.9, 55.4, 48.2, 41.5, 24.9, 23.2, 22.9.

*4-Hydroxy-6-methoxy-2,2-dimethyl-4-((trimethylsilyl)ethynyl)-3,4-dihydronaphthalen-1(2H)-one* (**6e**): *n*-BuLi (2.1 mL, 5 mmol, 2.4 mol/L) was added dropwise to a solution of ethynyltrimethylsilane (0.49 g, 5 mmol) in THF (10 mL) at −78 °C under a N_2_ atmosphere, and then the mixture was stirred for 1 h at –78 °C. A solution of **4c** (1.09 g, 5 mmol) in THF (5 mL) was added slowly, the mixture was stirred for 0.5 h at −78 °C, and then it was warmed to room temperature and stirred for 2 h. The reaction was quenched with saturated aqueous NH_4_Cl solution (10 mL) and extracted with EtOAc (3 × 30 mL). The combined organics were washed with brine (2 × 20 mL), dried over anhydrous Na_2_SO_4_ and concentrated. The residue was purified by silica gel column chromatography (petroleum ether/EtOAc, 6:1) to afford **6e** (1.39 g, 88%) as a red-brown liquid. ^1^H-NMR (CDCl_3_, 25 °C) δ 7.84 (d, *J* = 8.5 Hz, 1H), 7.48 (d, *J* = 2.7 Hz, 1H), 7.16 (dd, *J*_1_ = 2.7 Hz, *J*_2_ = 8.6 Hz, 1H), 3.85 (s, 3H), 2.92 (d, *J* = 16.6 Hz, 1H), 2.56 (d, *J* = 16.6 Hz, 1H), 2.53 (s, 1H), 1.22 (s, 3H), 1.13 (s, 3H), 0.20 (s, 9H). ^13^C-NMR (CDCl_3_, 25 °C) δ 197.1, 159.7, 131.2, 129.0, 121.5, 109.3, 105.4, 74.2, 55.5, 41.3, 24.8, −0.2. MS: 339.0 (M+Na^+^).

*Ethyl 3-(1′-hydroxy-7′-methoxy-3′,3′-dimethyl-4′-oxo-1′,2′,3′,4′-tetrahydronaphthalen-1′-yl)propiolate* (**6f**): LDA (2.5 mL, 5 mmol, 2.0 mol/L) was added dropwise to a solution of ethylpropiolate (0.49 g, 5 mmol) in THF (10 mL) at −78 °C under a N_2_ atmosphere, and then the mixture was stirred for 1 h at −78 °C. A solution of **4c** (1.09 g, 5 mmol) in THF (5 mL) was added slowly, the mixture was stirred for 0.5 h at −78 °C, and then it was warmed to room temperature and stirred for 2 h. The reaction was quenched with saturated aqueous NH_4_Cl solution (10 mL) and extracted with EtOAc (3 × 30 mL). The combined organics were washed with brine (2 × 20 mL), dried over anhydrous Na_2_SO_4_ and concentrated. The residue was purified by silica gel column chromatography (petroleum ether/EtOAc, 8:1) to afford **6f** (1.48 g, 94%) as a red-brown liquid. ^1^H-NMR (CDCl_3_, 25 °C) δ 7.79 (d, *J* = 8.6 Hz, 1H), 7.48 (d, *J* = 2.8 Hz, 1H), 7.15 (dd, *J*_1_ = 2.8 Hz, *J*_2_ = 8.6 Hz, 1H), 4.23 (q, *J*_1_ = 7.1 Hz, *J*_2_ = 14.2 Hz, 2H), 3.85 (s, 3H), 3.50 (d, *J* = 5.7 Hz, 1H), 2.92 (d, *J* = 17.1 Hz, 1H), 2.64 (d, *J* = 17.1 Hz, 1H), 1.31 (t, *J* = 7.1 Hz, 3H), 1.25 (s, 3H), 1.17 (s, 3H). ^13^C-NMR (CDCl_3_, 25 °C) δ 196.5, 160.0, 153.1, 134.7, 131.2, 129.0, 121.6, 109.7, 86.5, 78.9, 73.9, 62.2, 55.5, 41.6, 24.7, 23.3, 13.8. MS:339.0 (M+Na^+^).

*Methyl 3-(1′-hydroxy-7′-methoxy-3′,3′-dimethyl-4′-oxo-1′,2′,3′,4′-tetrahydronaphthalen-1′-yl)propiolate* (**6g**): LDA (2.5 mL, 5 mmol, 2.0 mol/L) was added dropwise to a solution of methyl propiolate (0.42 g, 5 mmol) in THF (10 mL) at −78 °C under a N_2_ atmosphere, and then the mixture was stirred for 1 h at −78 °C. A solution of **4c** (1.09 g, 5 mmol) in THF (5 mL) was added slowly, the mixture was stirred for 0.5 h at −78 °C, and then it was warmed to room temperature and stirred for 2 h. The reaction was quenched with saturated aqueous NH_4_Cl solution (10 mL) and extracted with EtOAc (3 × 30 mL). The combined organics were washed with brine (2 × 20 mL), dried over anhydrous Na_2_SO_4_ and concentrated. The residue was purified by silica gel column chromatography (petroleum ether/EtOAc, 9:1) to afford **6g** (1.37 g, 91%) as a red-brown liquid. ^1^H-NMR (CDCl_3_, 25 °C) δ 7.78 (d, *J* = 8.6 Hz, 1H), 7.50 (d, *J* = 2.8 Hz, 1H), 7.16 (dd, *J*_1_ = 2.8 Hz, *J*_2_ = 8.6 Hz, 1H), 3.86 (s, 3H), 3.79 (s, 3H), 3.03 (s, 1H), 2.93 (d, *J* = 17.1 Hz, 1H), 2.60 (d, *J* = 17.1 Hz, 1H), 1.23 (s, 3H), 1.16 (s, 3H). ^13^C-NMR (CDCl_3_, 25 °C) δ 196.3, 160.2, 153.5, 134.5, 131.2, 128.9, 121.7, 109.8, 86.9, 78.7, 74.1, 55.6, 52.8, 48.0, 41.6, 24.7, 23.1. MS:325.0 (M+Na^+^).

*(Z)-7-Bromo-4-hydroxy-4-(5′-hydroxy-3-methylpent-3′-en-1′-yn-1′-yl)-2,2-dimethyl-3,4-dihydronaphthalen-1(2H)-one* (**6h**): *n*-BuLi (4.2 mL, 10 mmol, 2.4 mol/L) was added dropwise to a solution of (*Z*)-3-methylpent-2-en-4-yn-1-ol (0.48 g, 5 mmol) in THF (10 mL) at −78 °C under a N_2_ atmosphere, and then the mixture was stirred for 1 h at −78 °C. A solution of **4d** (1.33 g, 5 mmol) in THF (5 mL) was added slowly, the mixture was stirred for 0.5 h at −78 °C, and then it was warmed to room temperature and stirred for 2 h. The reaction was quenched with saturated aqueous NH_4_Cl solution (10 mL) and extracted with EtOAc (3 × 30 mL). The combined organics were washed with brine (2 × 20 mL), dried over anhydrous Na_2_SO_4_ and concentrated. The residue was purified by silica gel column chromatography (petroleum ether/EtOAc, 2:1) to afford **6h** (1.61 g, 89%) as a yellow oil. ^1^H-NMR (CDCl_3_, 25 °C) δ 8.06 (d, *J* = 1.8 Hz, 1H), 7.85 (d, *J* = 8.3 Hz, 1H), 7.55 (dd, *J*_1_ = 1.9 Hz, *J*_2_ = 8.3 Hz, 1H), 5.93–5.89 (m, 1H), 4.26 (d, *J* = 6.7 Hz, 2H), 4.03 (s, 1H), 2.81 (d, *J* = 24.1 Hz, 1H), 2.64 (d, *J* = 24.1 Hz, 1H), 2.59 (s, 1H), 1.88 (s, 3H), 1.16 (s, 6H). ^13^C-NMR (CDCl_3_, 25 °C) δ 196.6, 146.1, 136.6, 131.7, 130.4, 129.4, 128.7, 128.5, 120.1, 93.6, 86.3, 74.0, 60.8, 48.5, 41.5, 29.5, 24.8, 22.8, 22.7.

*(Z)-5-Hydroxy-5-(5′-hydroxy-3′-methylpent-3′-en-1′-yn-1′-yl)-2,7,7-trimethyl-6,7-dihydroquinolin-8(5H)-one* (**6i**): *n*-BuLi (4.2 mL, 10 mmol, 2.4 mol/L) was added dropwise to a solution of (*Z*)-3-methylpent-2-en-4-yn-1-ol (0.48 g, 5 mmol) in THF (10 mL) at −78 °C under a N_2_ atmosphere, and then the mixture was stirred for 1 h at −78 °C. A solution of **4e** (1.01 g, 5 mmol) in THF (5 mL) was added slowly, the mixture was stirred for 0.5 h at −78 °C, and then it was warmed to room temperature and stirred for 2 h. The reaction was quenched with saturated aqueous NH_4_Cl solution (10 mL) and extracted with EtOAc (3 × 30 mL). The combined organics were washed with brine (2 × 20 mL), dried over anhydrous Na_2_SO_4_ and concentrated. The residue was purified by silica gel column chromatography (petroleum ether/EtOAc, 1:1) to afford **6i** (1.40 g, 94%) as a yellow oil. ^1^H-NMR (CDCl_3_, 25 °C) δ 8.17 (d, *J* = 7.9 Hz, 1H), 7.28 (d, *J* = 7.9 Hz, 1H), 5.85–5.83 (m, 1H), 5.34 (s, 1H), 4.16 (d, *J* = 6.5 Hz, 2H), 2.99 (d, *J* = 17.5 Hz, 1H), 2.59 (d, *J* = 17.5 Hz, 1H), 2.65 (s, 3H), 1.79 (s, 3H), 1.39 (s, 3H), 0.96 (s, 3H). ^13^C-NMR (CDCl_3_, 25 °C) δ 195.8, 163.5, 160.8, 136.8, 135.4, 123.4, 122.5, 119.4, 94.3, 85.2, 73.6, 60.8, 50.2, 40.7, 24.6, 24.6, 22.6, 20.9.

*(Z)-5-Hydroxy-5-(5′-hydroxy-3-methylpent-3′-en-1′-yn-1′-yl)-7,7-dimethyl-2-phenyl-6,7-dihydroquinolin-8(5H)-one* (**6j**): *n*-BuLi (4.2 mL, 10 mmol, 2.4 mol/L) was added dropwise to a solution of (*Z*)-3-methylpent-2-en-4-yn-1-ol (0.48 g, 5 mmol) in THF (10 mL) at −78 °C under a N_2_ atmosphere, and then the mixture was stirred for 1 h at −78 °C. A solution of **4f** (1.32 g, 5 mmol) in THF (5 mL) was added slowly, the mixture was stirred for 0.5 h at −78 °C, and then it was warmed to room temperature and stirred for 2 h. The reaction was quenched with saturated aqueous NH_4_Cl solution (10 mL) and extracted with EtOAc (3 × 30 mL). The combined organics were washed with brine (2 × 20 mL), dried over anhydrous Na_2_SO_4_ and concentrated. The residue was purified by silica gel column chromatography (petroleum ether/EtOAc, 1:1) to afford **6j** (1.73 g, 96%) as a yellow oil. ^1^H-NMR (CDCl_3_, 25 °C) δ 8.35 (d, *J* = 8.1 Hz, 1H), 8.11 (dd, *J*_1_ = 2.7 Hz, *J*_2_ = 7.9 Hz, 2H), 7.87 (d, *J* = 8.2 Hz, 1H), 7.55–7.51 (m, 3H), 5.84–5.79 (m, 1H), 5.33 (s, 1H), 4.16 (s, 2H), 3.07 (d, *J* = 17.5 Hz, 1H), 2.62 (d, *J* = 17.5 Hz, 1H), 1.80 (d, *J* = 1.1 Hz, 3H), 1.45 (s, 3H), 0.99 (s, 3H). ^13^C-NMR (CDCl_3_, 25 °C) δ 195.7, 161.3, 160.5, 137.3, 136.6, 136.3, 130.6, 129.0, 127.5, 123.4, 120.2, 119.8, 94.6, 85.3, 73.8, 61.2, 50.6, 40.9, 24.8, 22.8, 21.0.

*(Z)-5-Hydroxy-5-(5′-hydroxy-3-methylpent-3′-en-1′-yn-1′-yl)-7,7-dimethyl-6,7-dihydroquinolin-8(5H)-one* (**6k**): *n*-BuLi (4.2 mL, 10 mmol, 2.4 mol/L) was added dropwise to a solution of (*Z*)-3-methylpent-2-en-4-yn-1-ol (0.48 g, 5 mmol) in THF (10 mL) at −78 °C under a N_2_ atmosphere, and then the mixture was stirred for 1 h at −78 °C. A solution of **4g** (0.94 g, 5 mmol) in THF (5 mL) was added slowly, the mixture was stirred for 0.5 h at −78 °C, and then it was warmed to room temperature and stirred for 2 h. The reaction was quenched with saturated aqueous NH_4_Cl solution (10 mL) and extracted with EtOAc (3 × 30 mL). The combined organics were washed with brine (2 × 20 mL), dried over anhydrous Na_2_SO_4_ and concentrated. The residue was purified by silica gel column chromatography (petroleum ether/EtOAc, 1:2) to afford **6c** (1.35 g, 95%) as a yellow oil. ^1^H-NMR (CDCl_3_, 25 °C) δ 8.77 (dd, *J*_1_ = 1.7 Hz, *J*_2_ = 4.8 Hz, 1H), 8.30 (dd, *J*_1_ = 1.7 Hz, *J*_2_ = 7.8 Hz, 1H), 7.45 (dd, *J*_1_ = 4.8 Hz, *J*_2_ = 7.8 Hz, 1H), 5.87–5.82 (m, 1H), 5.48 (s, 1H), 4.14 (d, *J* = 6.5 Hz, 2H), 3.00 (d, *J* = 17.4 Hz, 1H), 2.68 (d, *J* = 17.4 Hz, 1H), 3.02 (s, 1H), 1.78 (d, *J* = 1.2 Hz, 3H), 1.40 (d, *J* = 6.8 Hz, 3H), 1.01 (s, 3H). ^13^C-NMR (CDCl_3_, 25 °C) δ 196.0, 161.4, 153.1, 136.9, 135.2, 124.9, 123.6, 119.3, 93.9, 85.7, 73.9, 60.6, 50.0, 40.7, 24.6, 22.6, 21.1.

## 4. Conclusions

We have developed a novel regioselective addition of acetylides to enediones that affords the corresponding 1-ethynylcyclohex-2-enol derivatives with excellent regioselectivity and high yield. This synthetic strategy can be applied to the manufacture of pharmaceuticals, fine chemicals, and natural products. Applications of this strategy in natural products are currently being carried out in our laboratory and will be reported in due course.

## Supplementary Materials

Supplementary materials can be accessed at: http://www.mdpi.com/1420-3049/18/9/10776/s1.
