# Supplementary material for: A Highly Efficient Regioselective Addition of Acetylides to Enediones Based on Steric Effects
Source: Molecules. 2013 Sep 3;18(9):10776–88. doi: 10.3390/molecules180910776 (PMC6270633; doi:10.3390/molecules180910776)

Supplementary Materials

Figure S1. ^{1}H-NMR of compound **3a**.

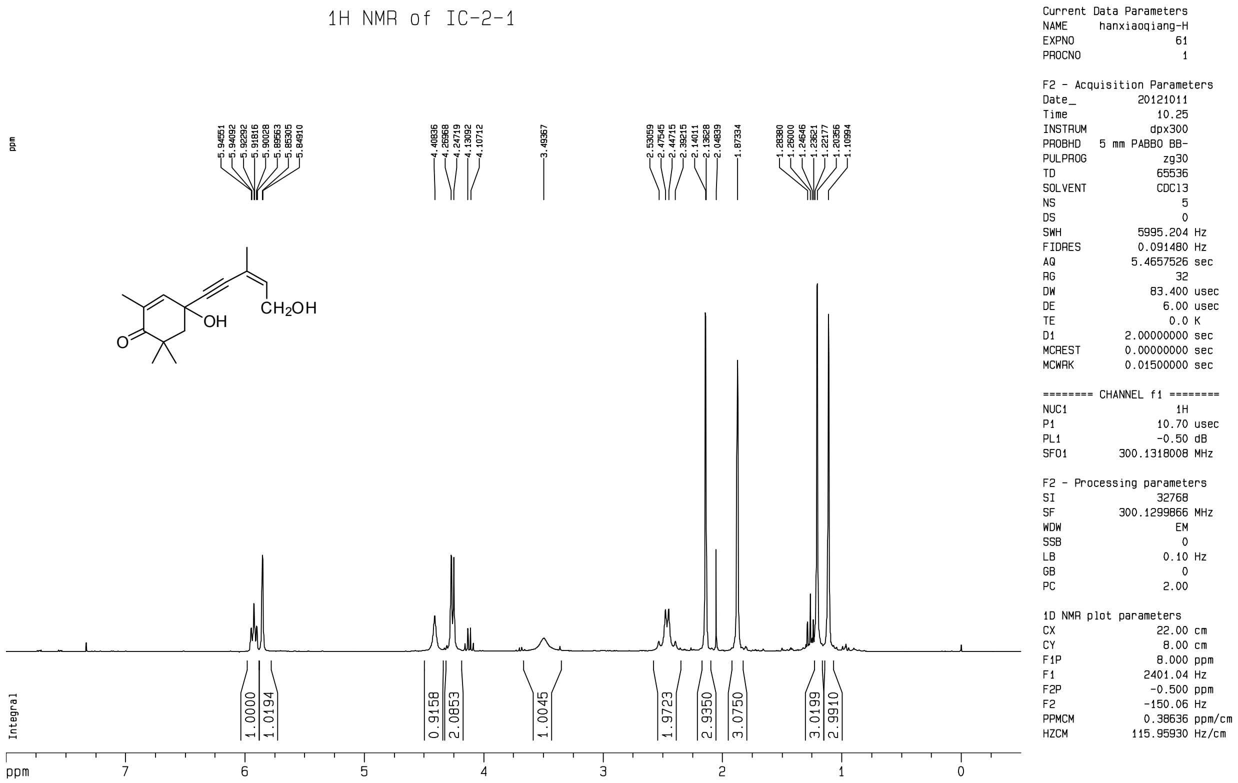

Figure S2. ^{13}C-NMR of compound **3a**.

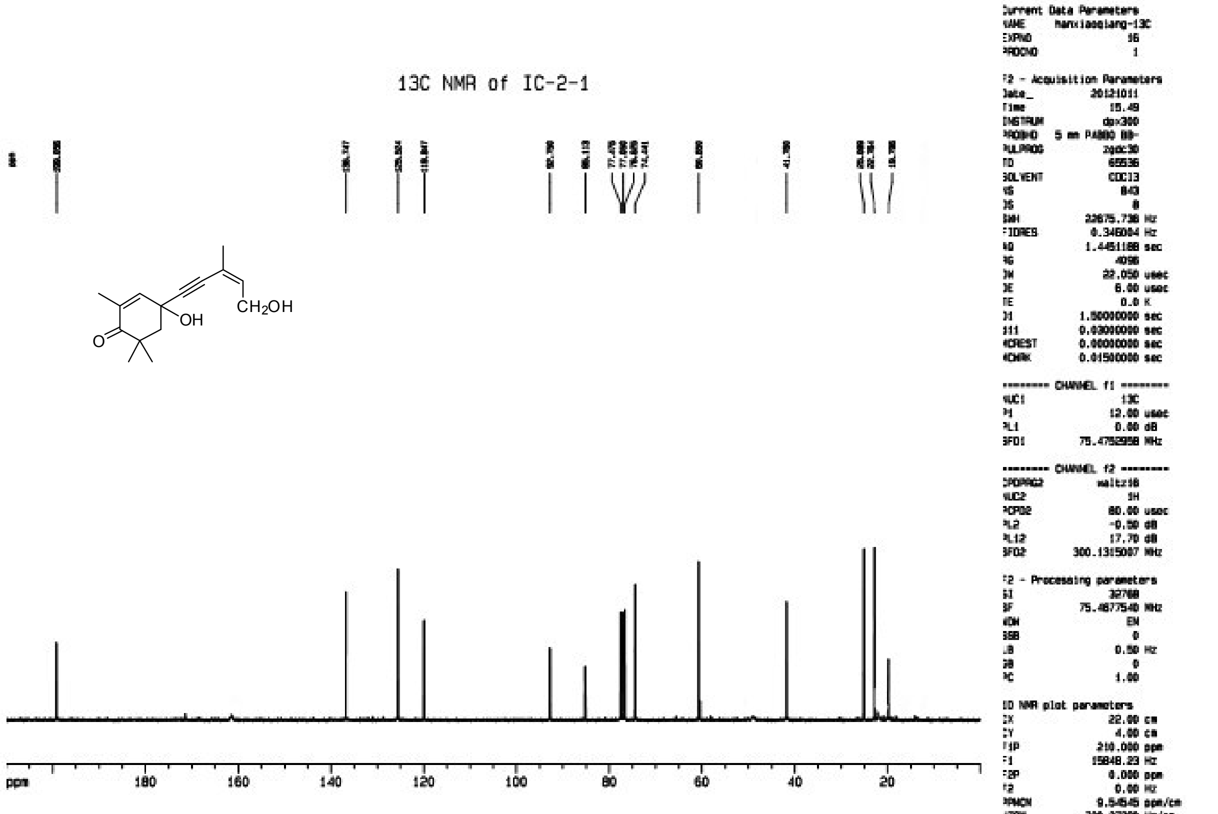

Figure S3. ^{1}H-NMR of compound **3b**.

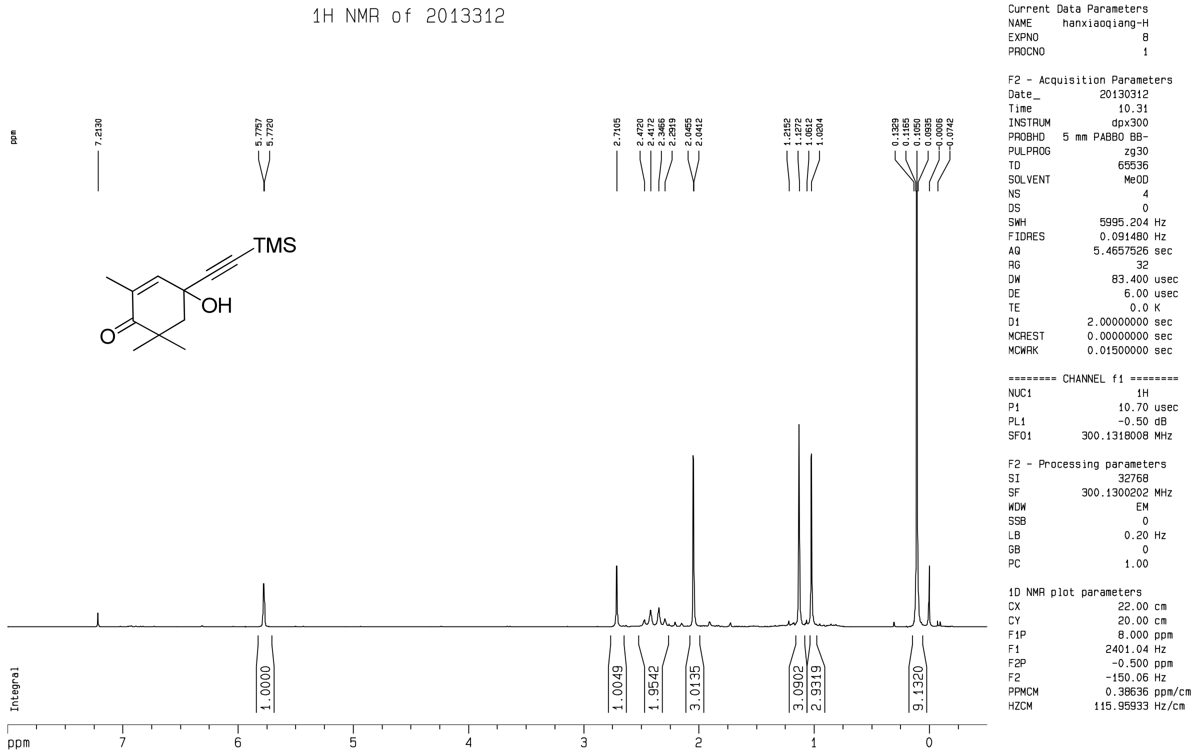

Figure S4. ^{13}C-NMR of compound **3b**.

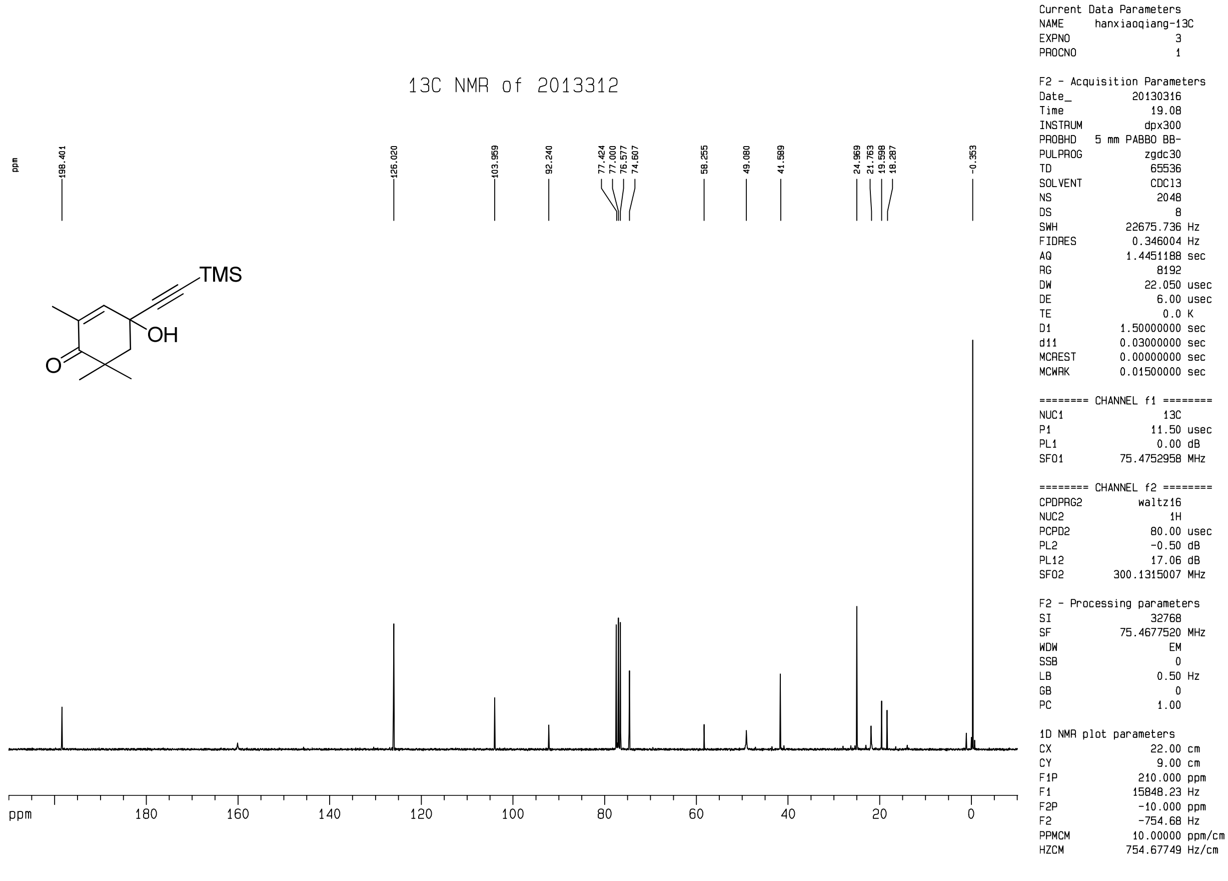

Figure S5. MS of compound **3b** (M+Na).

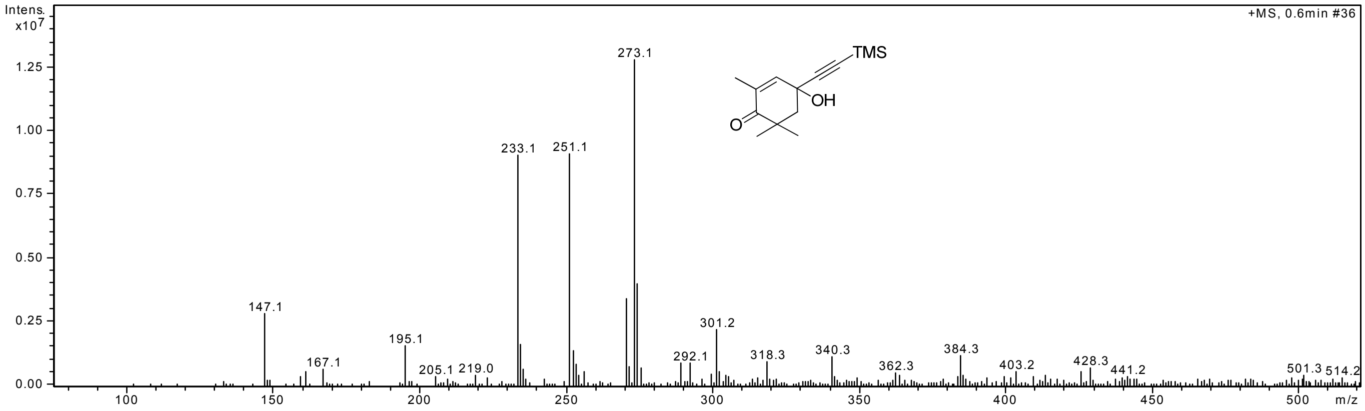

Figure S6. ^{1}H-NMR of compound **3c**.

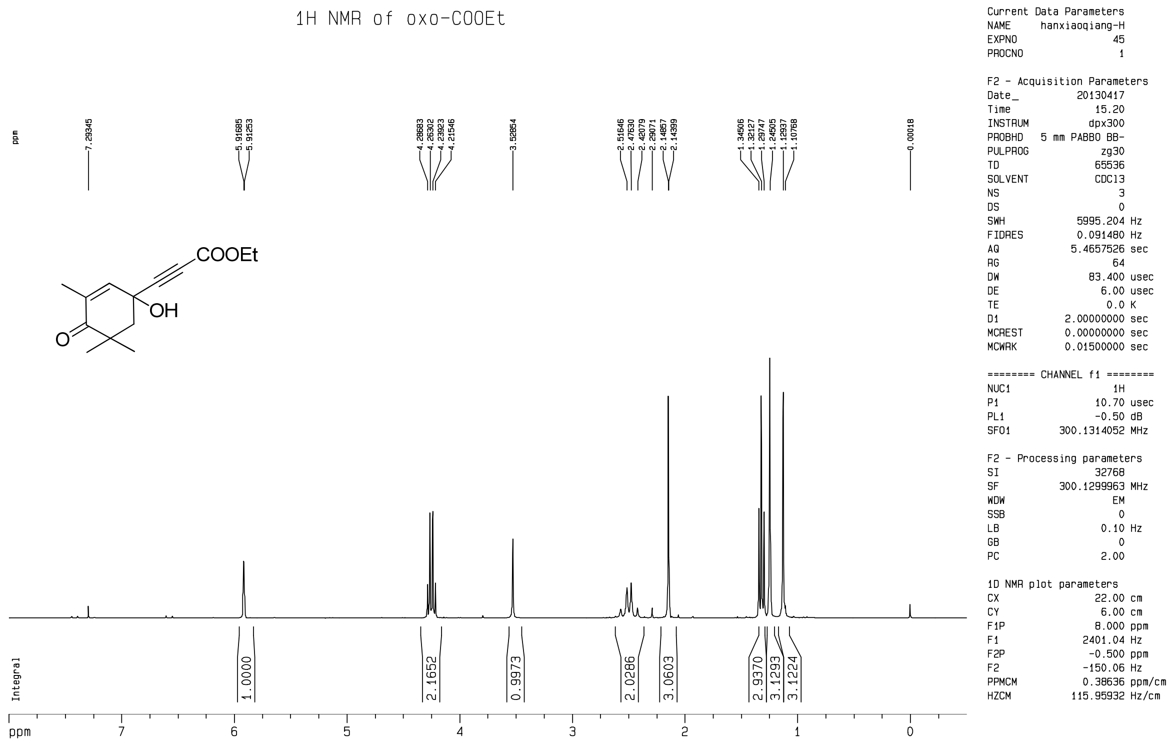

Figure S7. ^{13}C-NMR of compound **3c**.

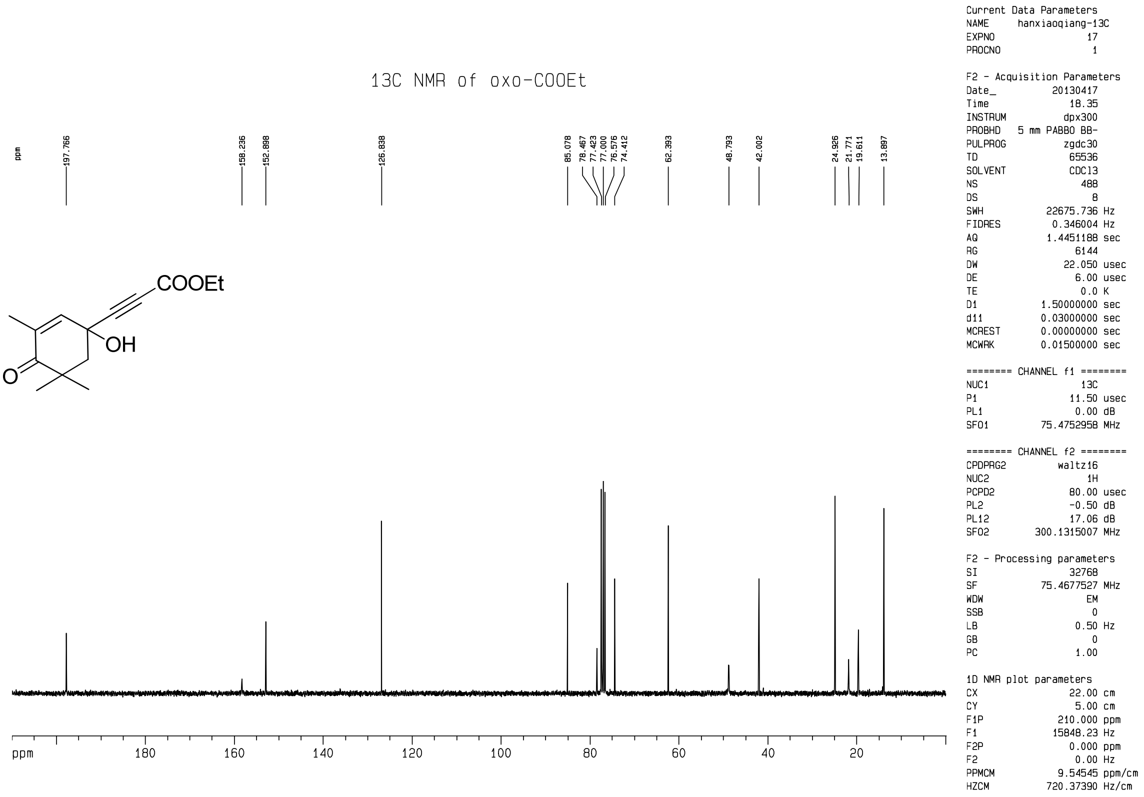

Figure S8. MS of compound **3c** (M+Na).

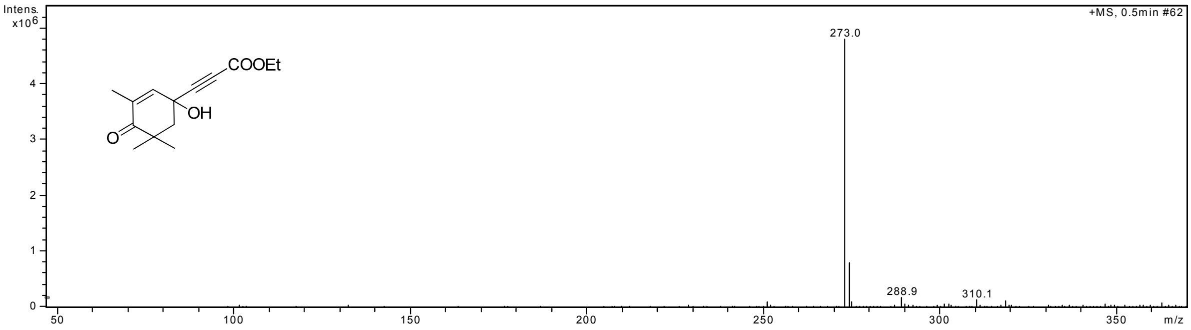

Figure S9. ^{1}H-NMR of compound **3d**.

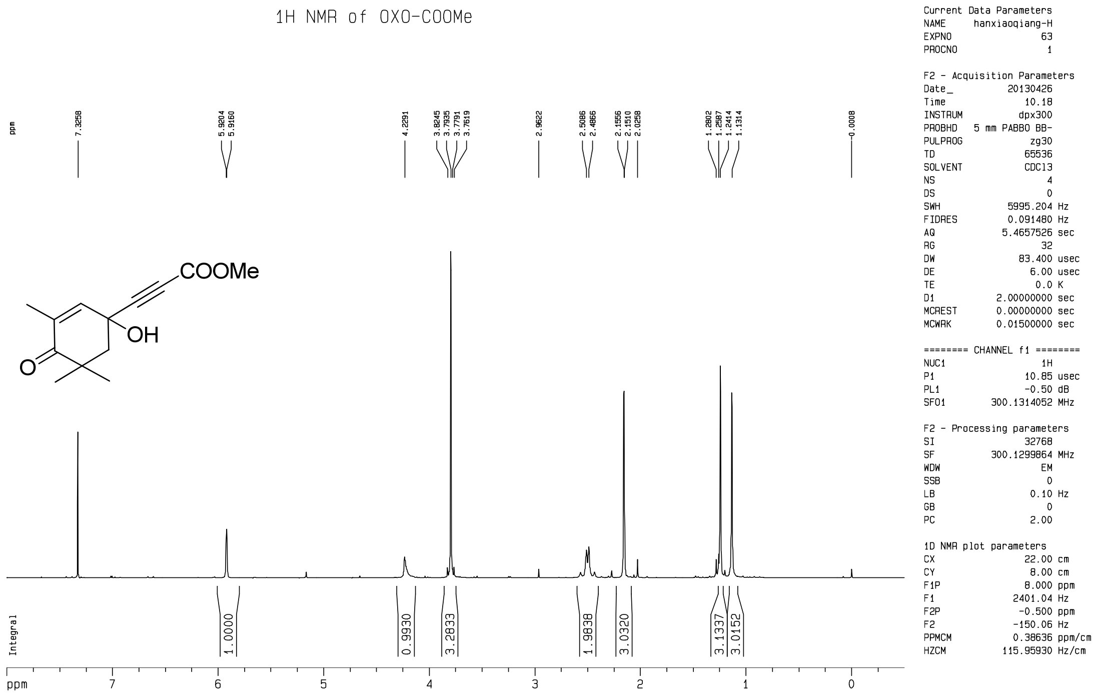

Figure S10. ^{13}C-NMR of compound **3d**.

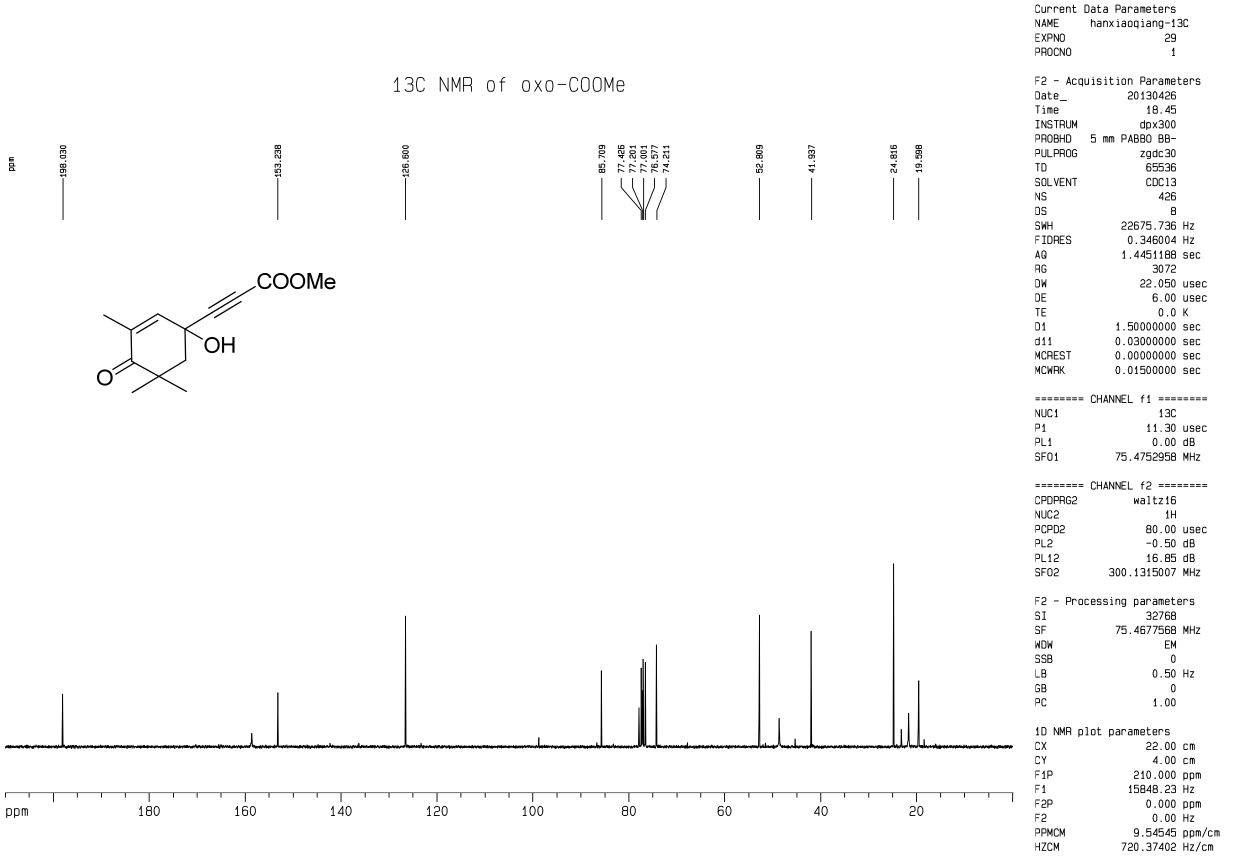

Figure S11. MS of compound **3d** (M+Na).

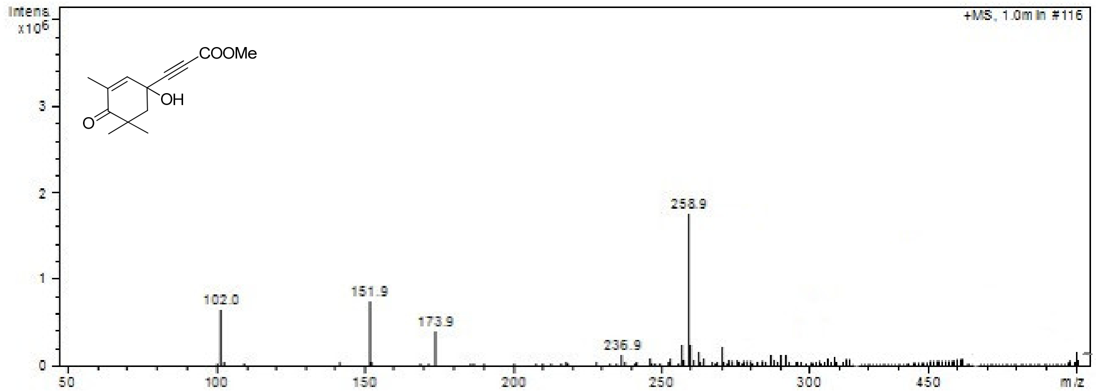

Figure S12. ^{1}H-NMR of compound **3e**.

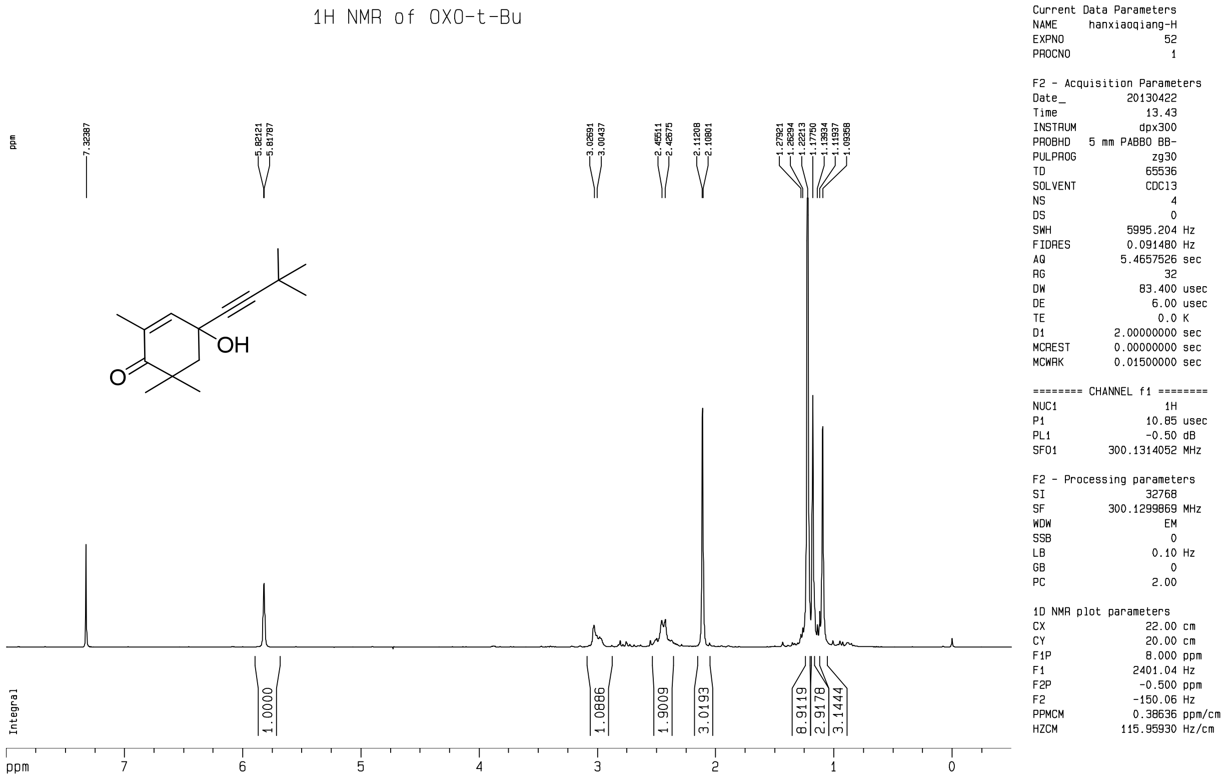

Figure S13. ^{13}C-NMR of compound **3e**.

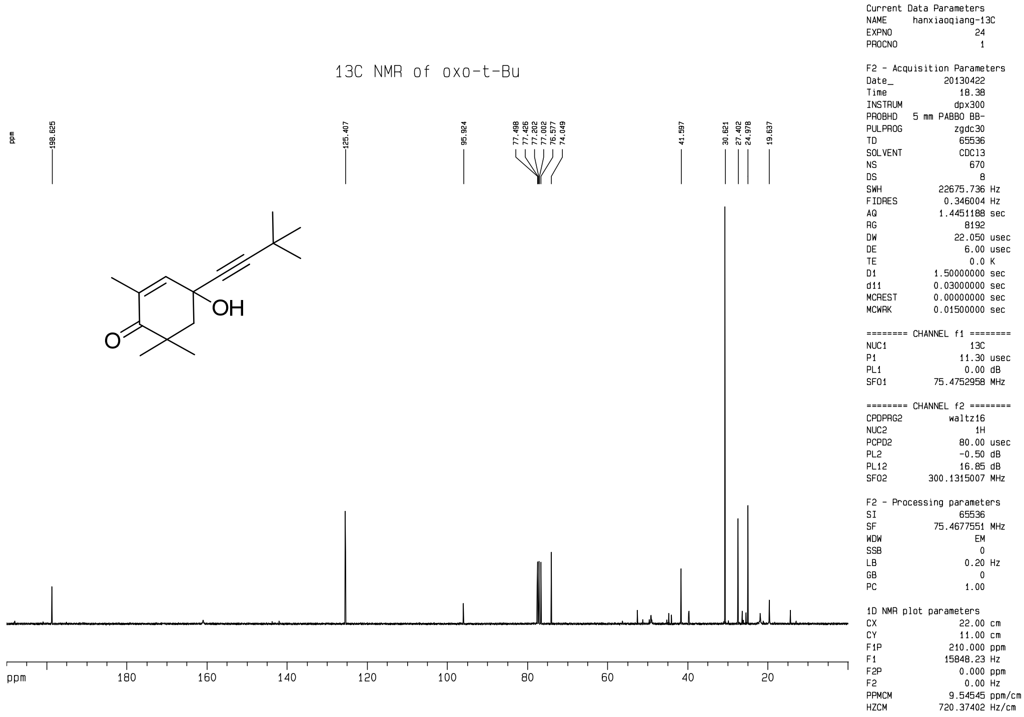

Figure S14. MS of compound **3e** (M+Na).

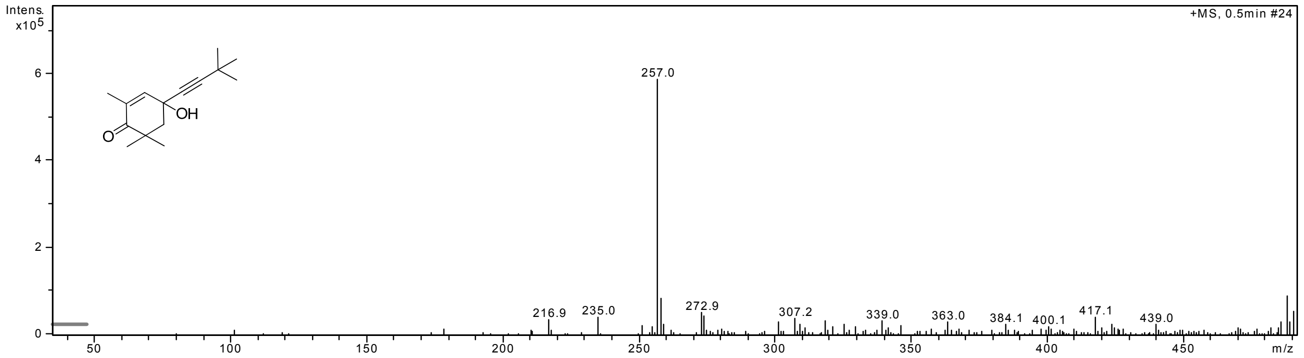

Figure S15. ^{1}H-NMR of compound **3f**.

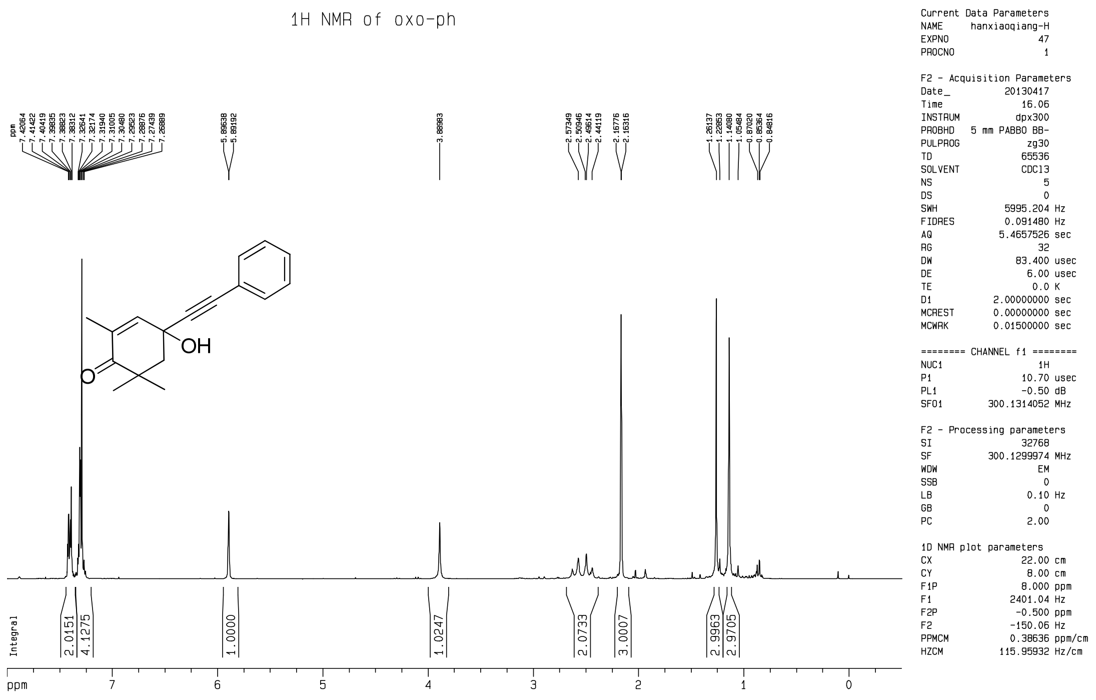

Figure S16. ^{13}C-NMR of compound **3f**.

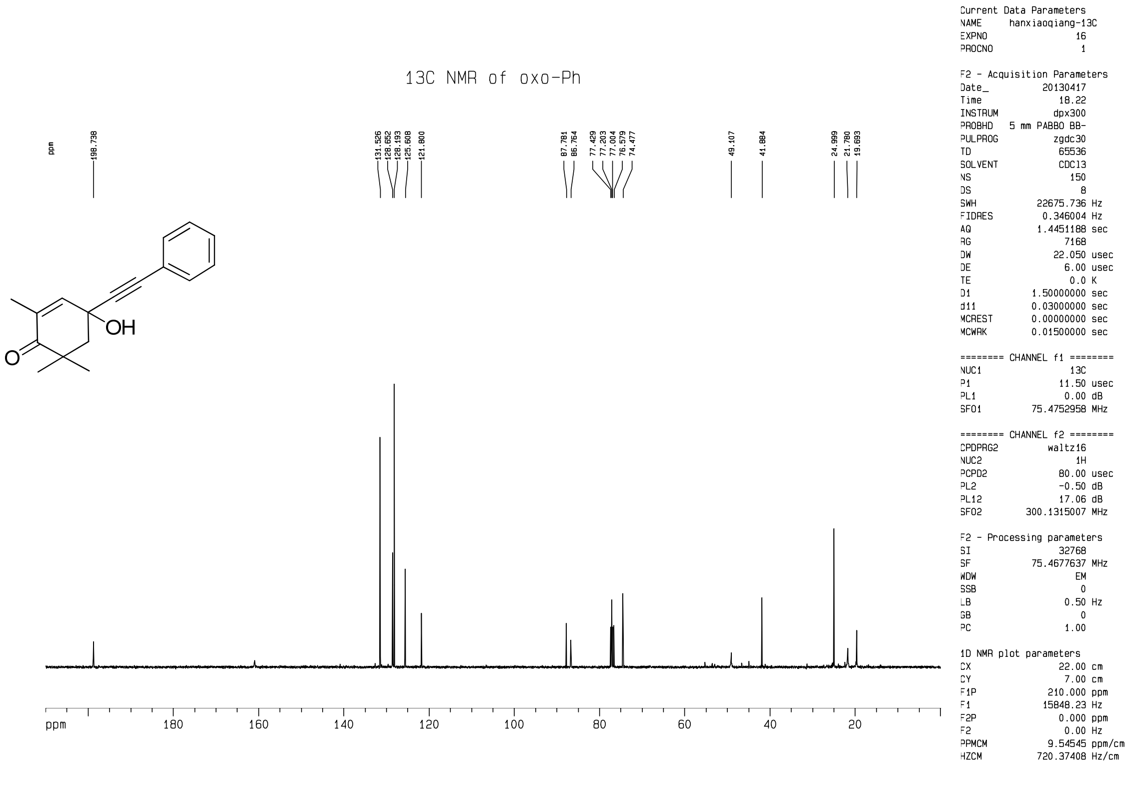

Figure S17. MS of compound **3f** (M+Na).

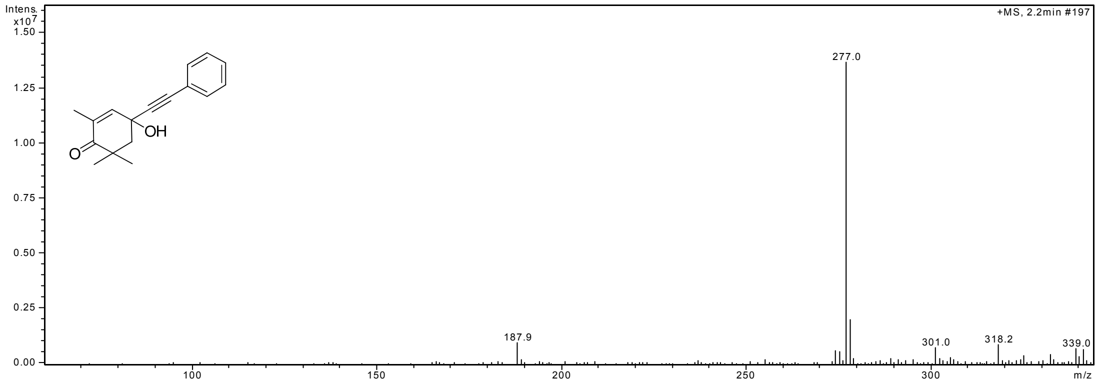

Figure S18. ^{1}H-NMR of compound **6a**.

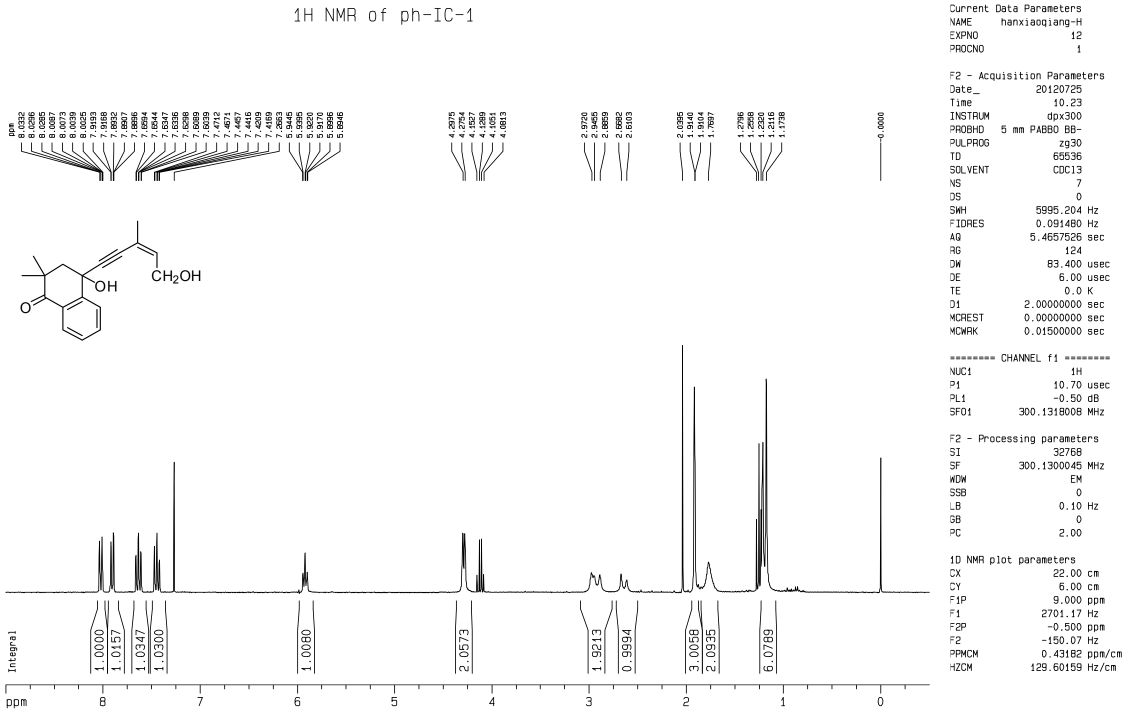

Figure S19. ^{13}C-NMR of compound **6a**.

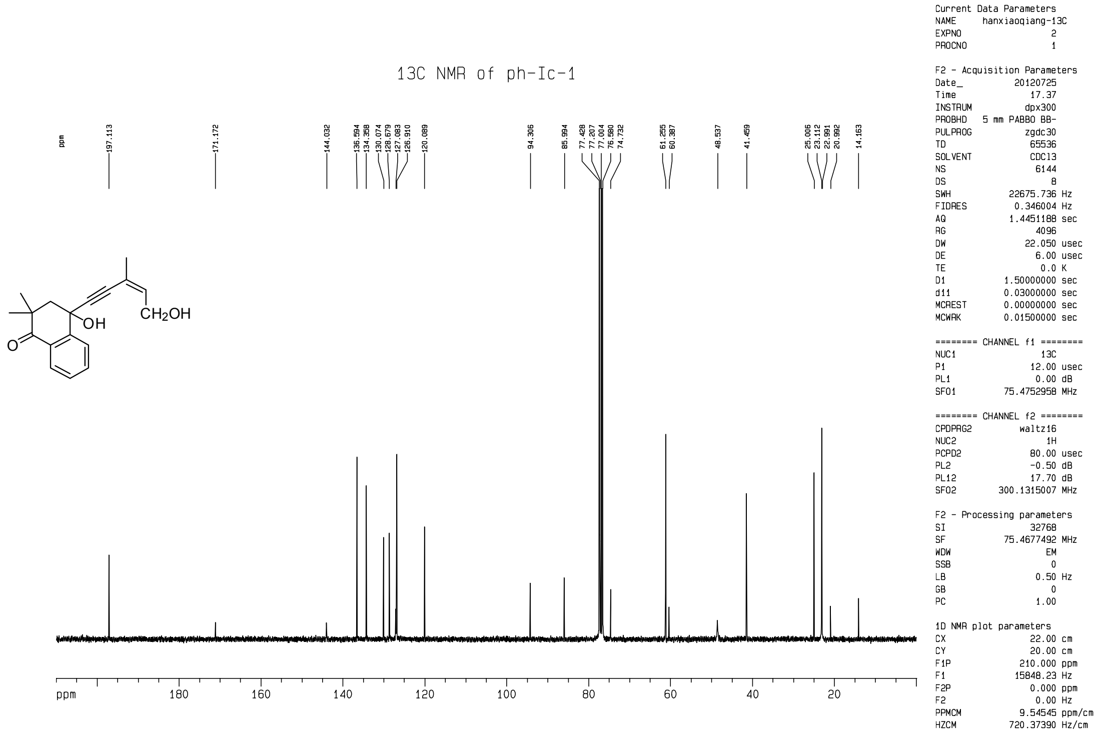

Figure S20. ^{1}H-NMR of compound **6b**.

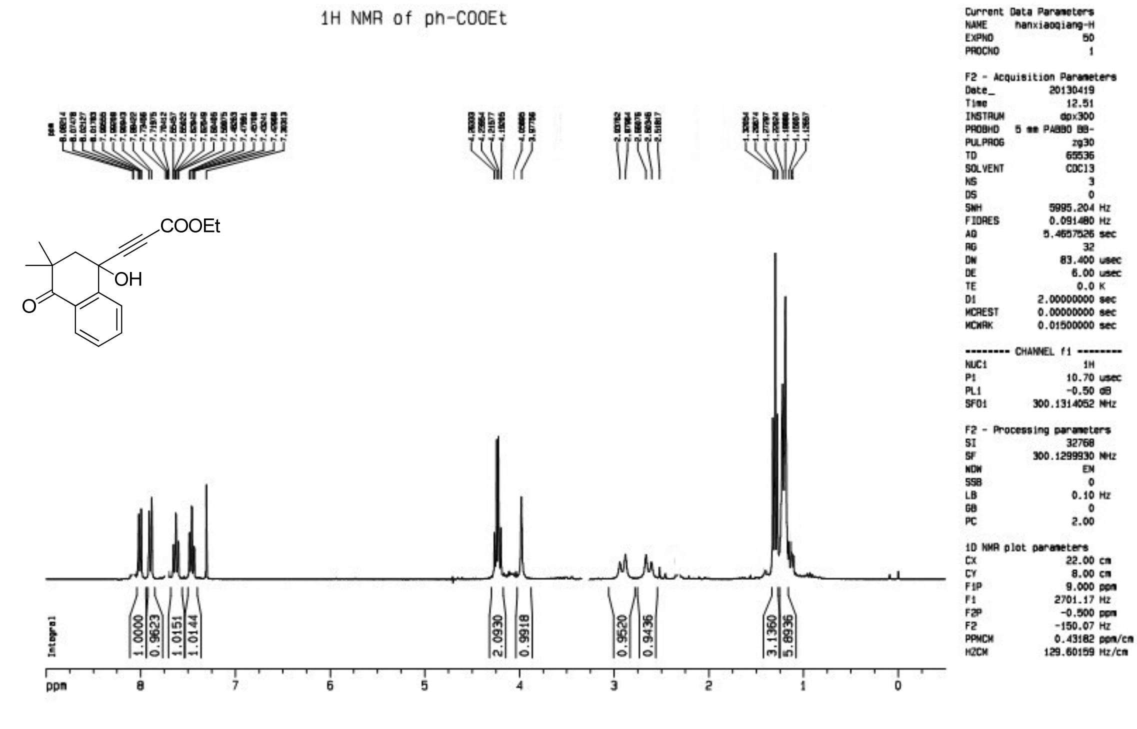

Figure S21. ^{13}C-NMR of compound **6b**.

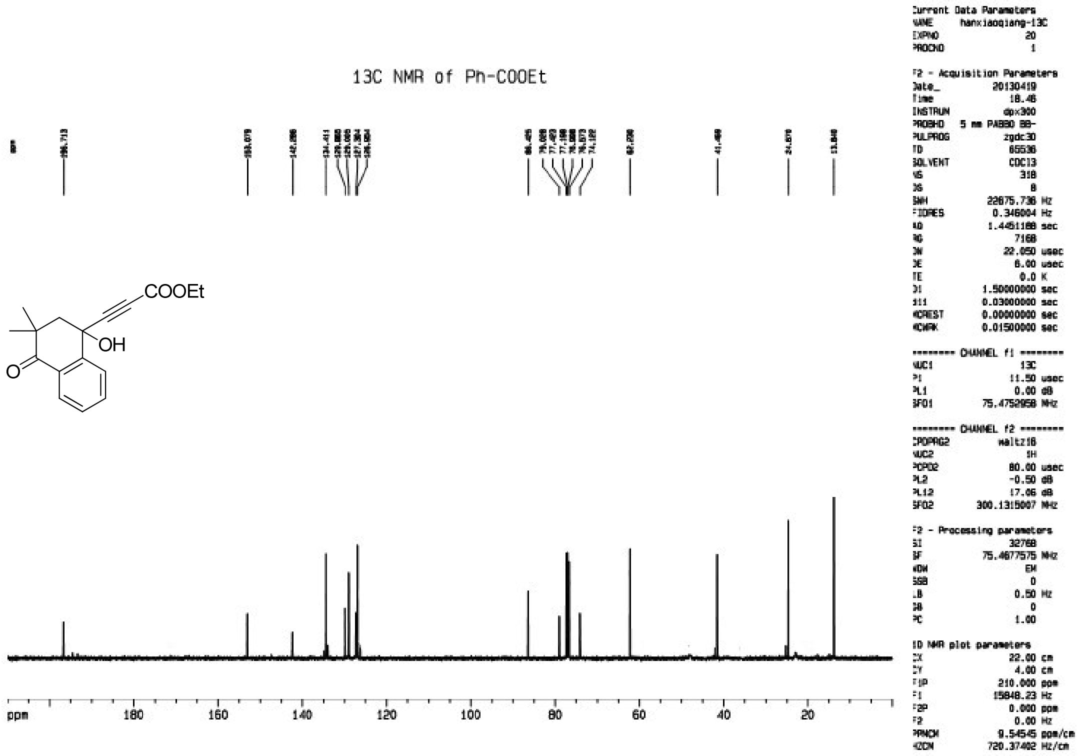

Figure S22. MS of compound **6b** (M+Na).

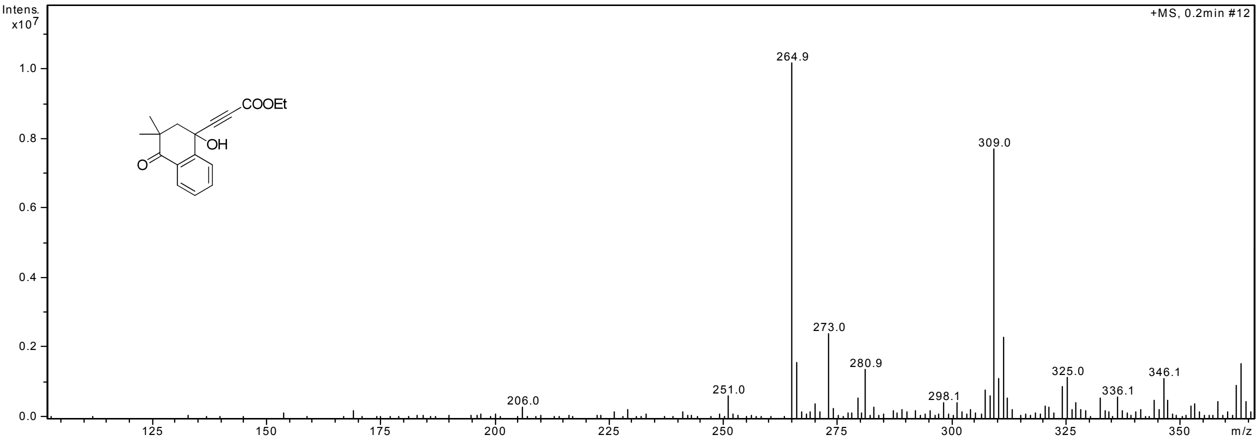

Figure S23. ^{1}H-NMR of compound **6c**.

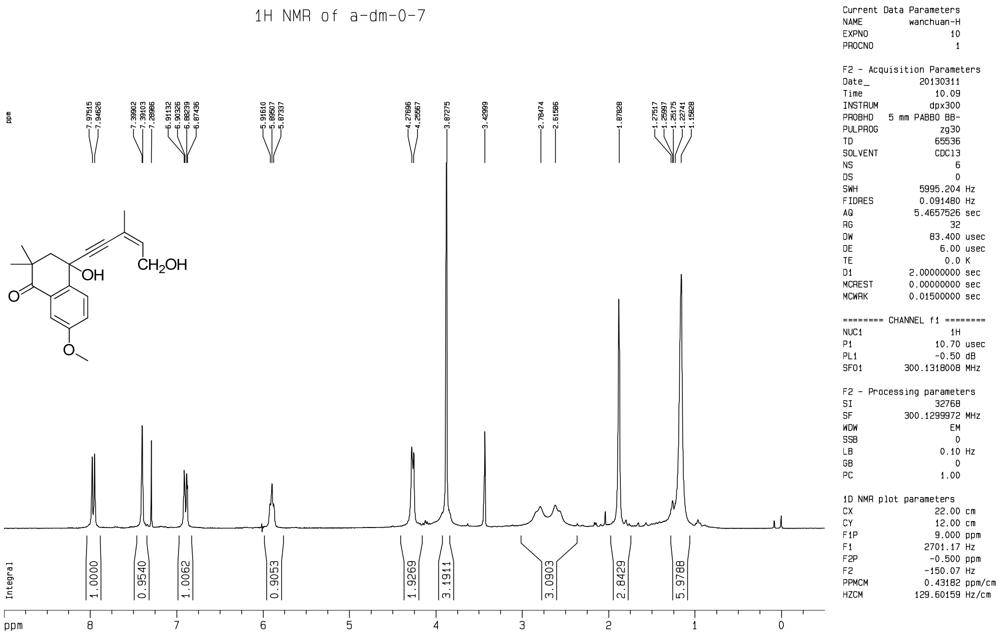

Figure S24. ^{13}C-NMR of compound **6c**.

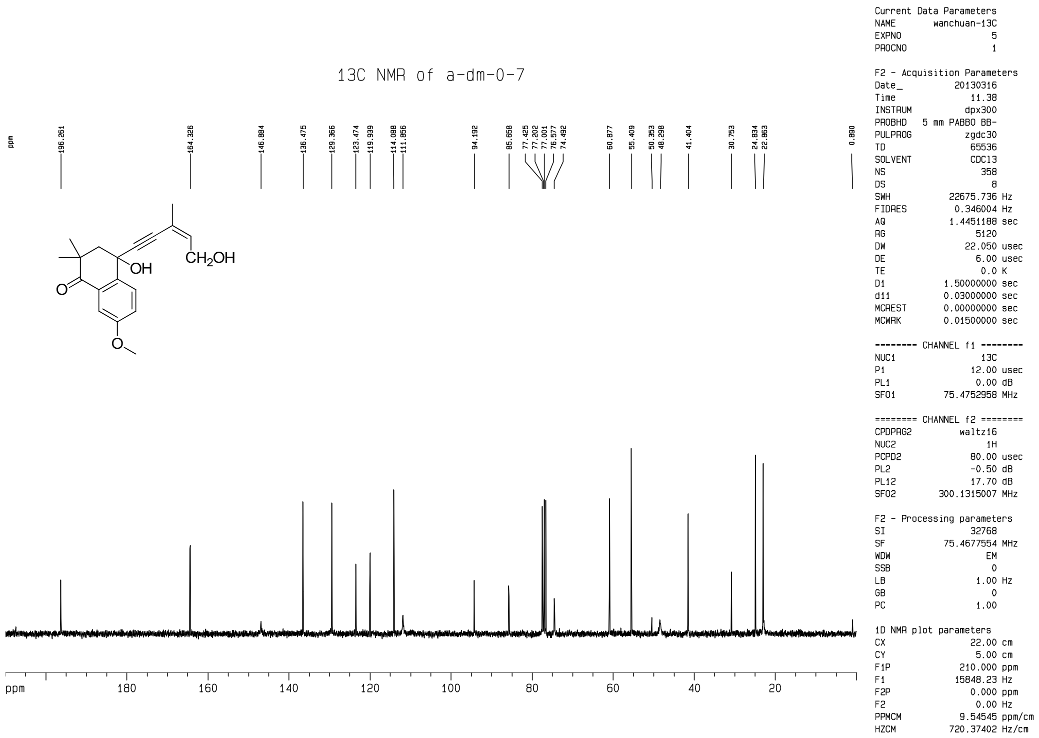

Figure S25. ^{1}H-NMR of compound **6d**.

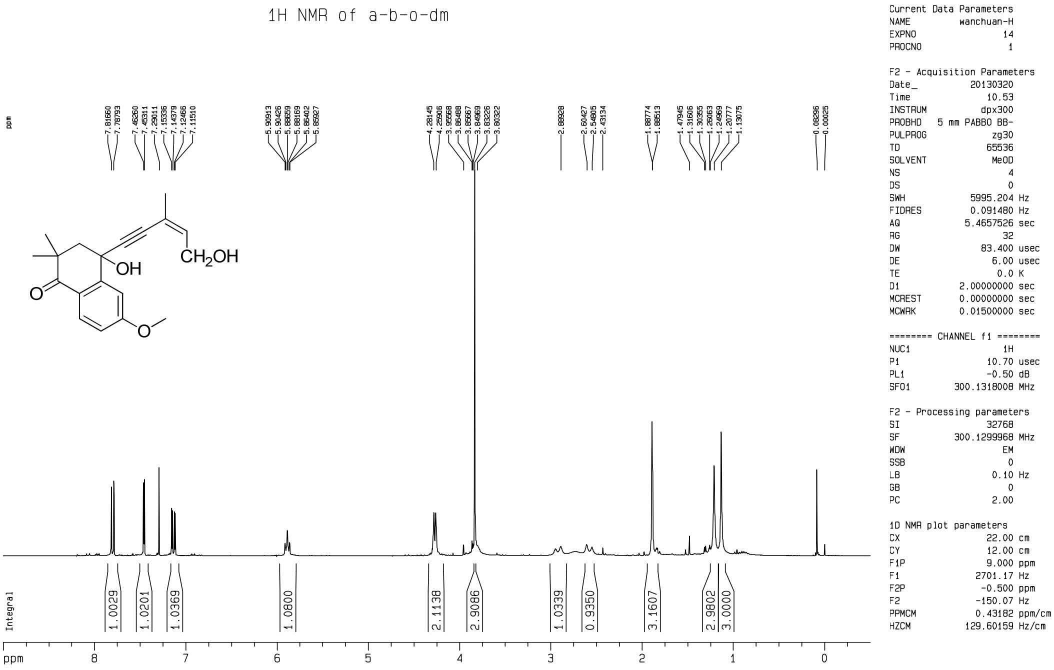

Figure S26. ^{13}C-NMR of compound **6d**.

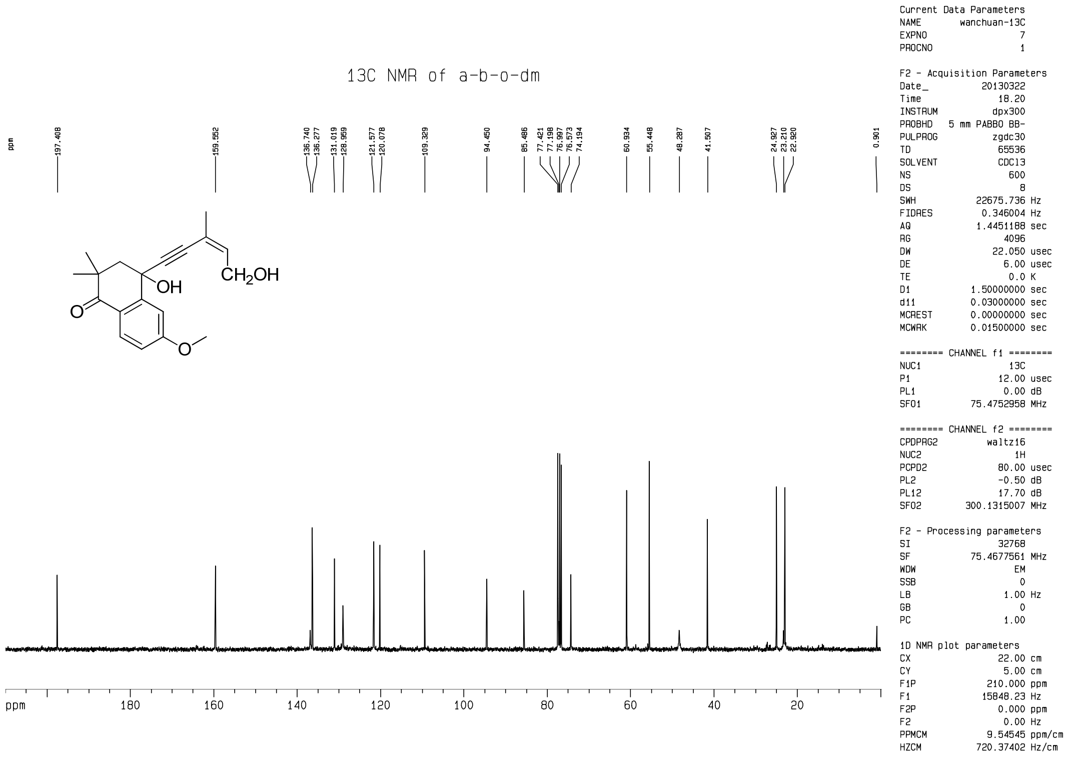

Figure S27. ^{1}H-NMR of compound **6e**.

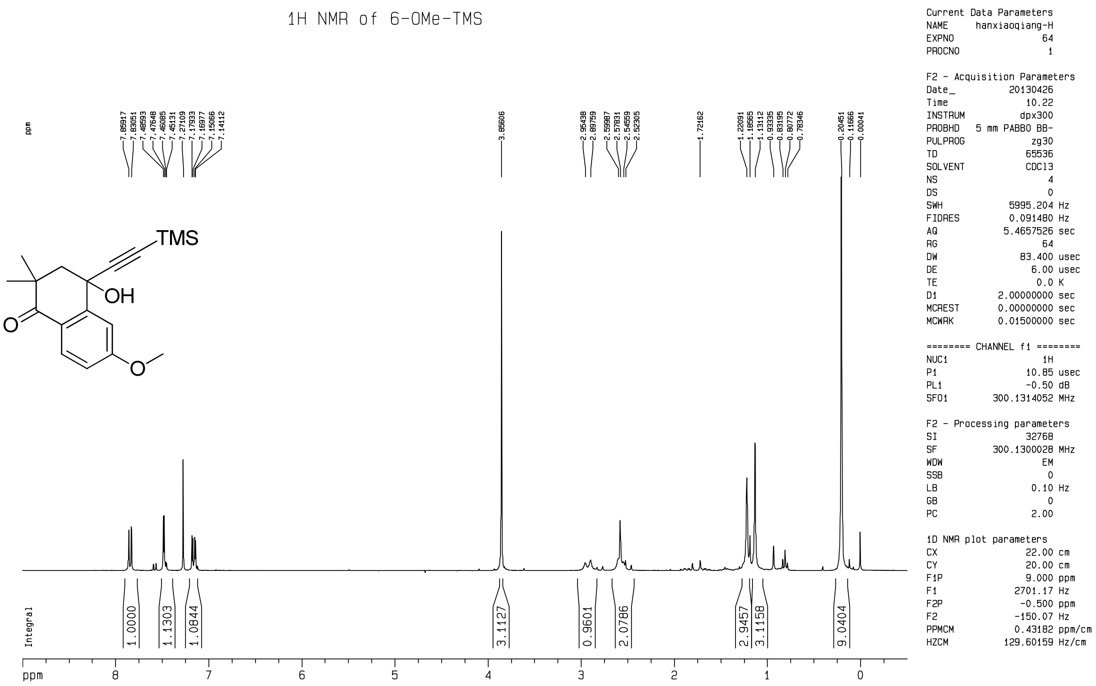

Figure S28. ^{13}C-NMR of compound **6e**.

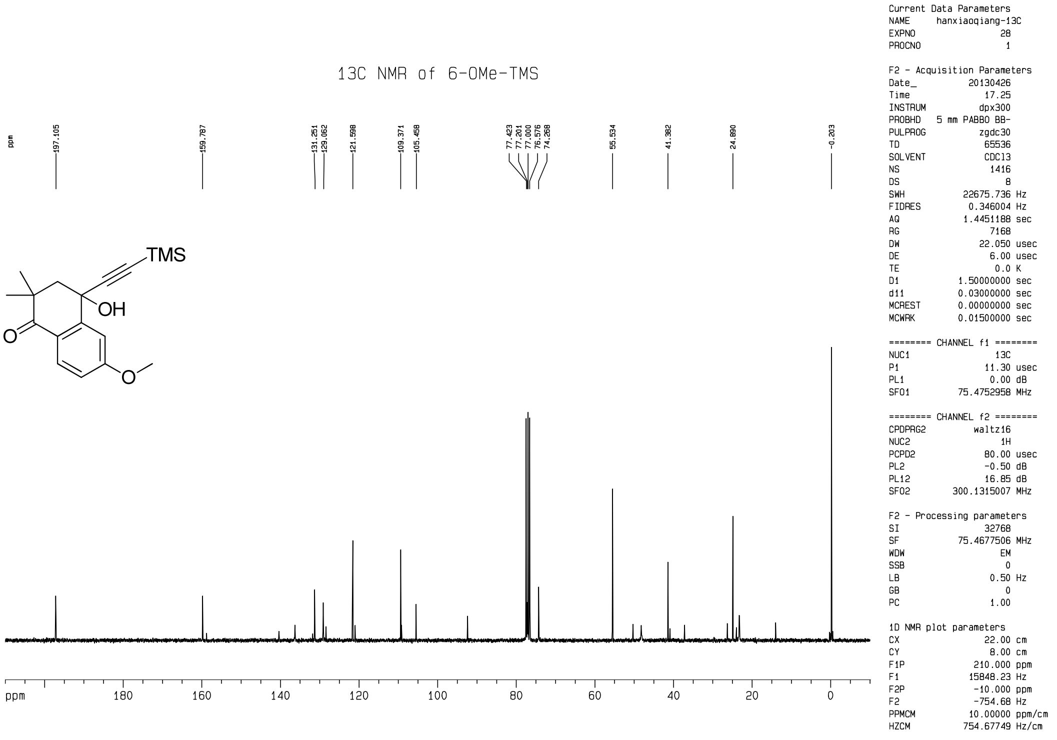

Figure S29. MS of compound **6e** (M+Na).

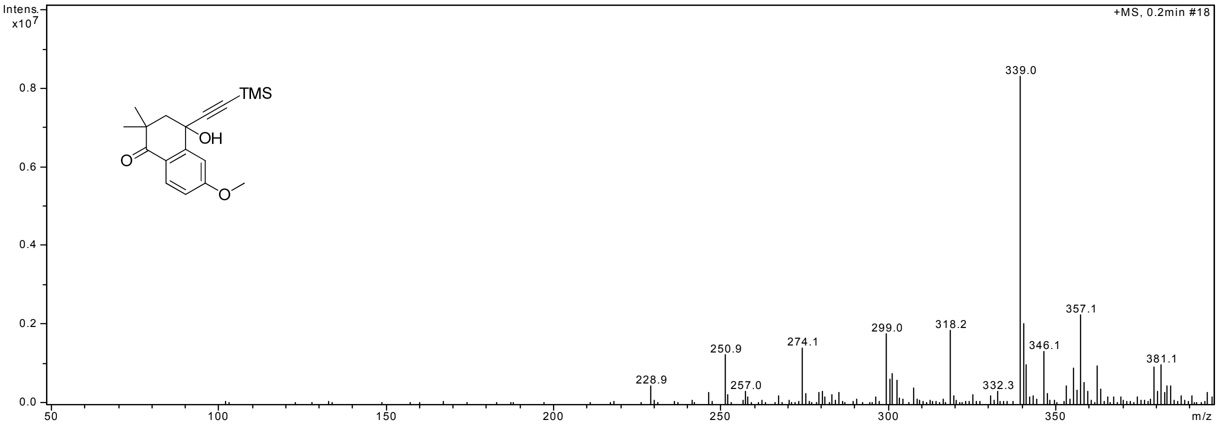

Figure S30. ^{1}H-NMR of compound **6f**.

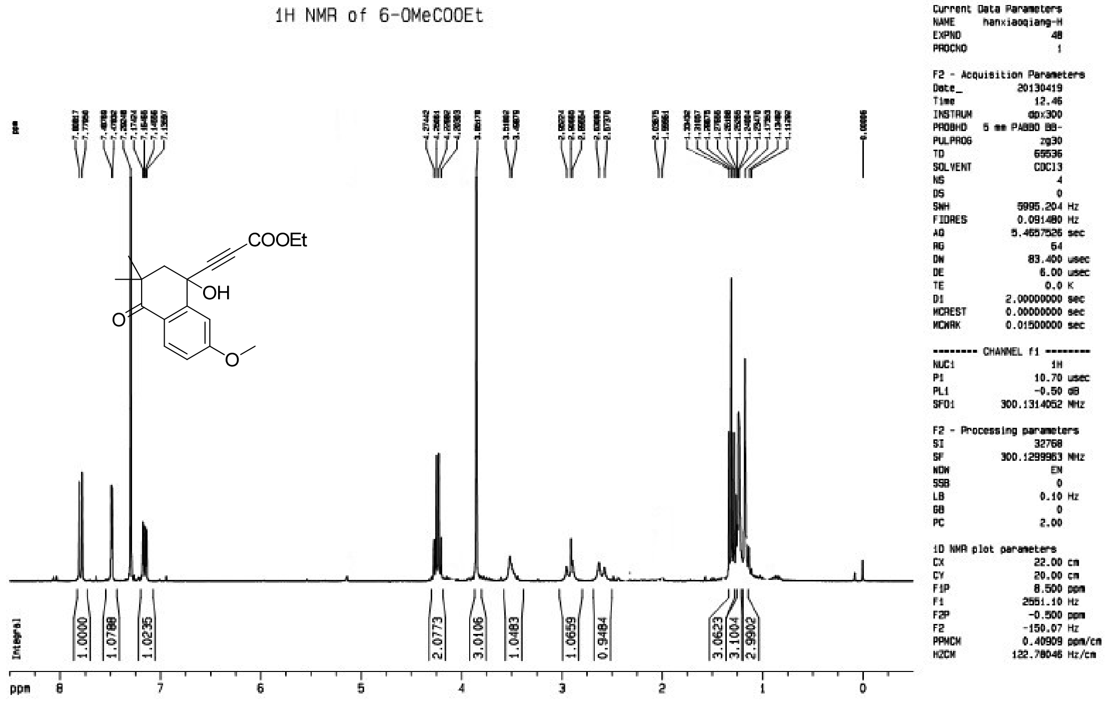

Figure S31. ^{13}C-NMR of compound **6f**.

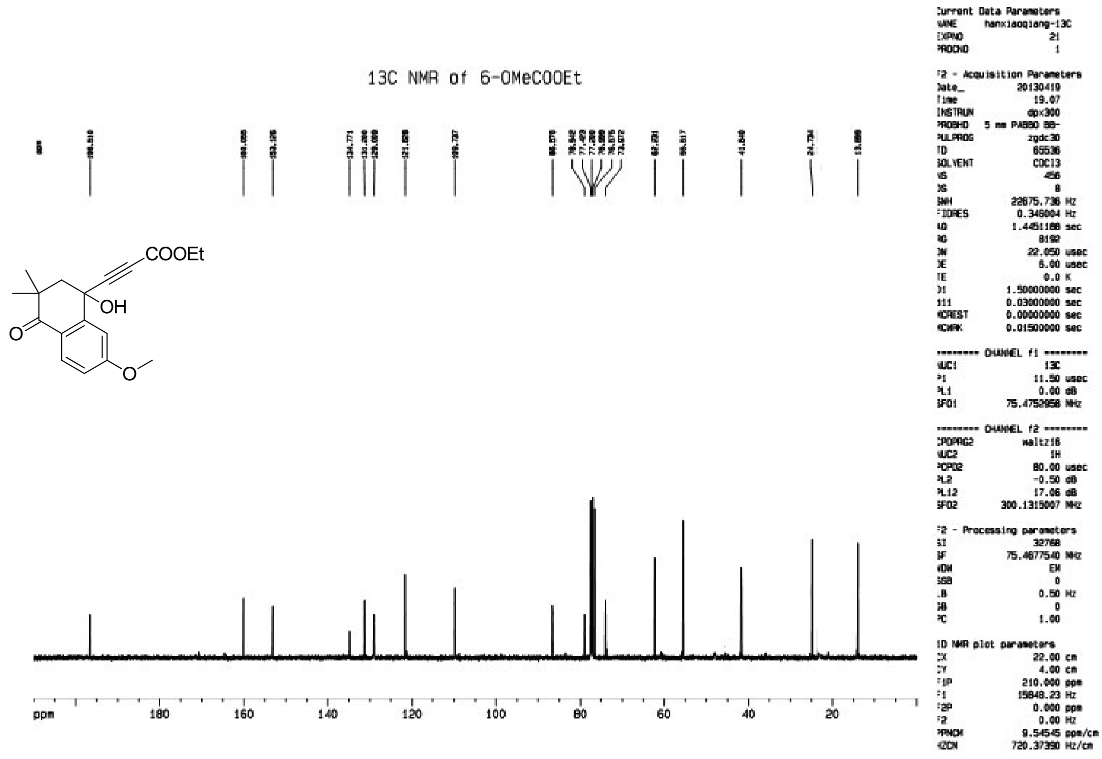

Figure S32. MS of compound **6f** (M+Na).

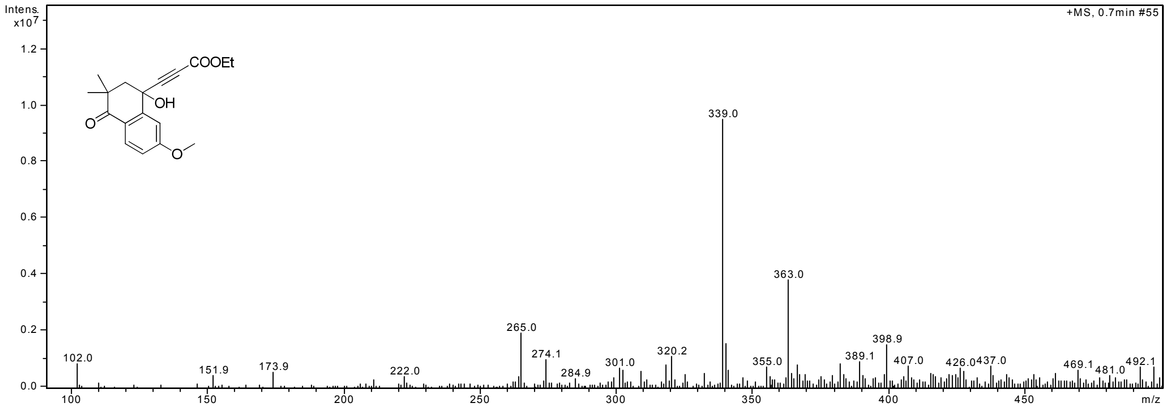

Figure S33. ^{1}H-NMR of compound **6g**.

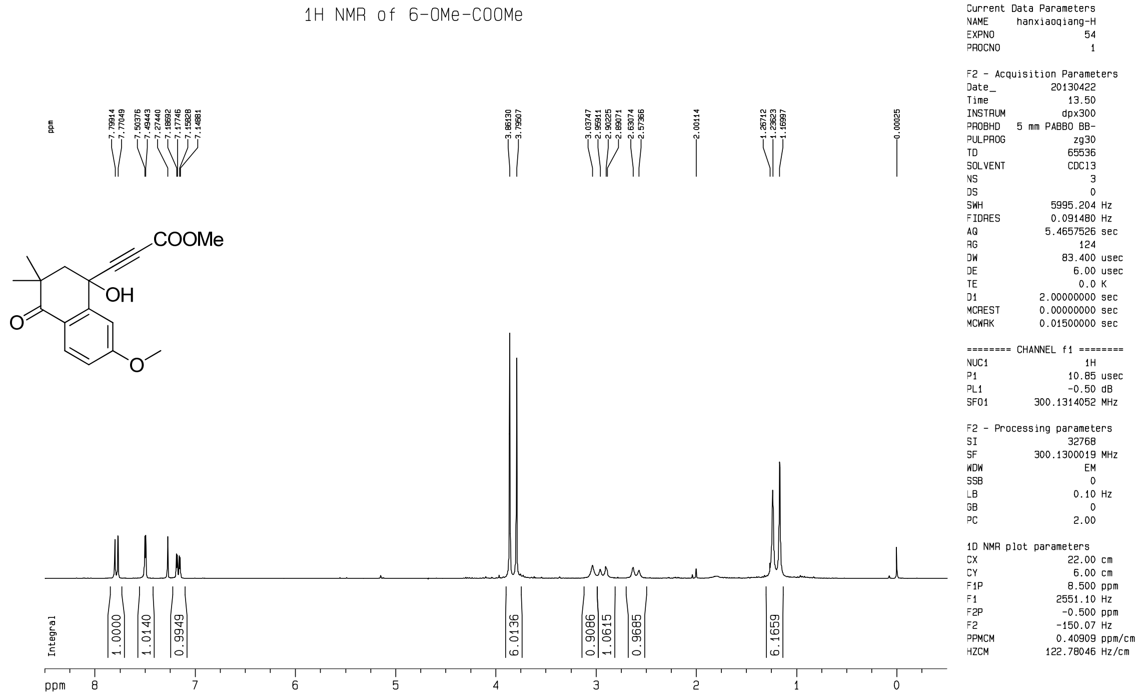

Figure S34. ^{13}C-NMR of compound **6g**.

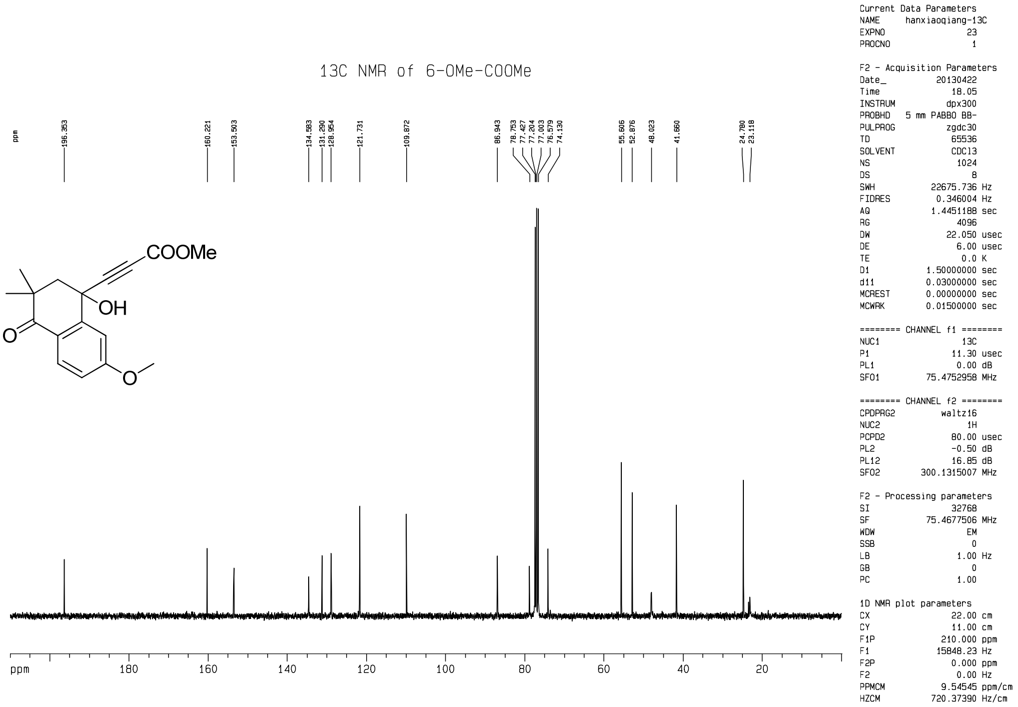

Figure S35. MS of compound **6g** (M+Na).

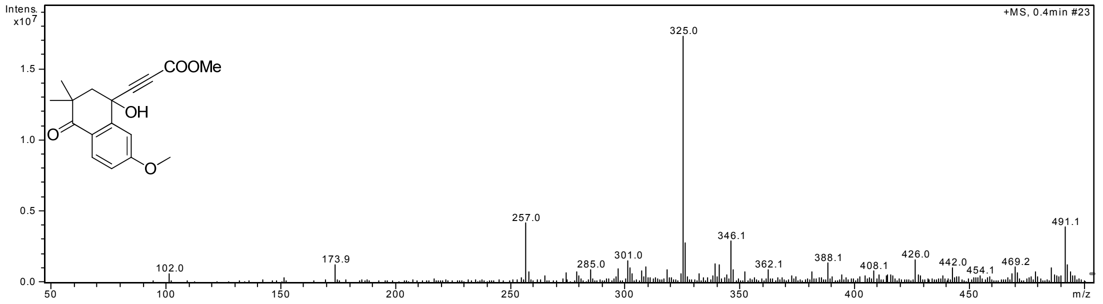

Figure S36. ^{1}H-NMR of compound **6h**.

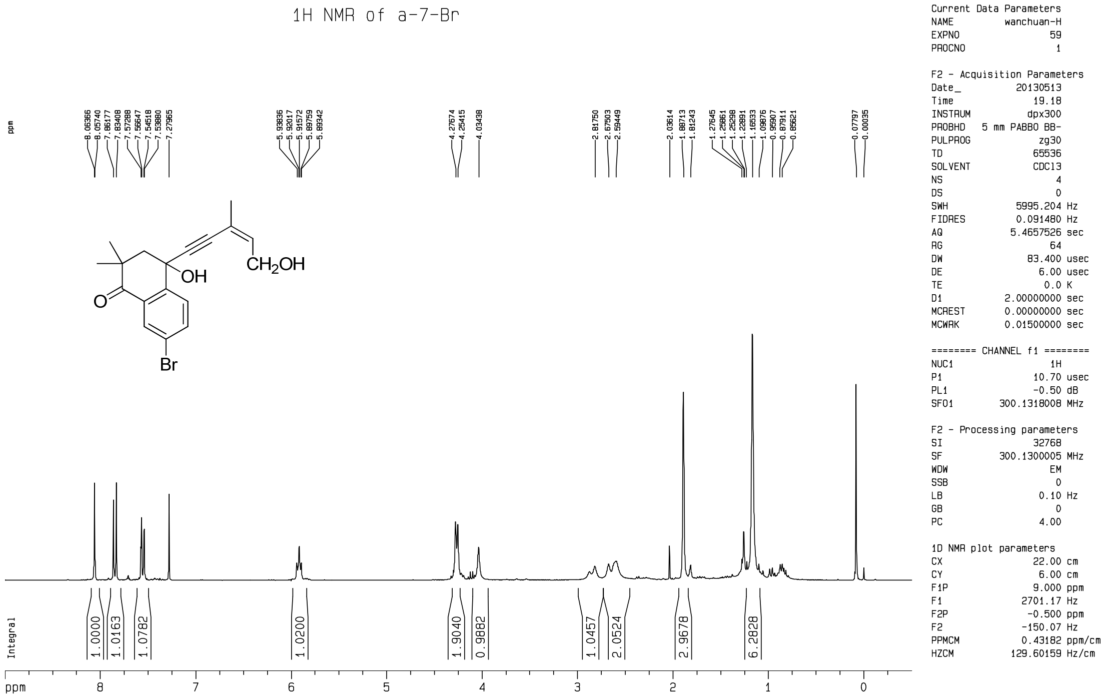

Figure S37. ^{13}C-NMR of compound **6h**.

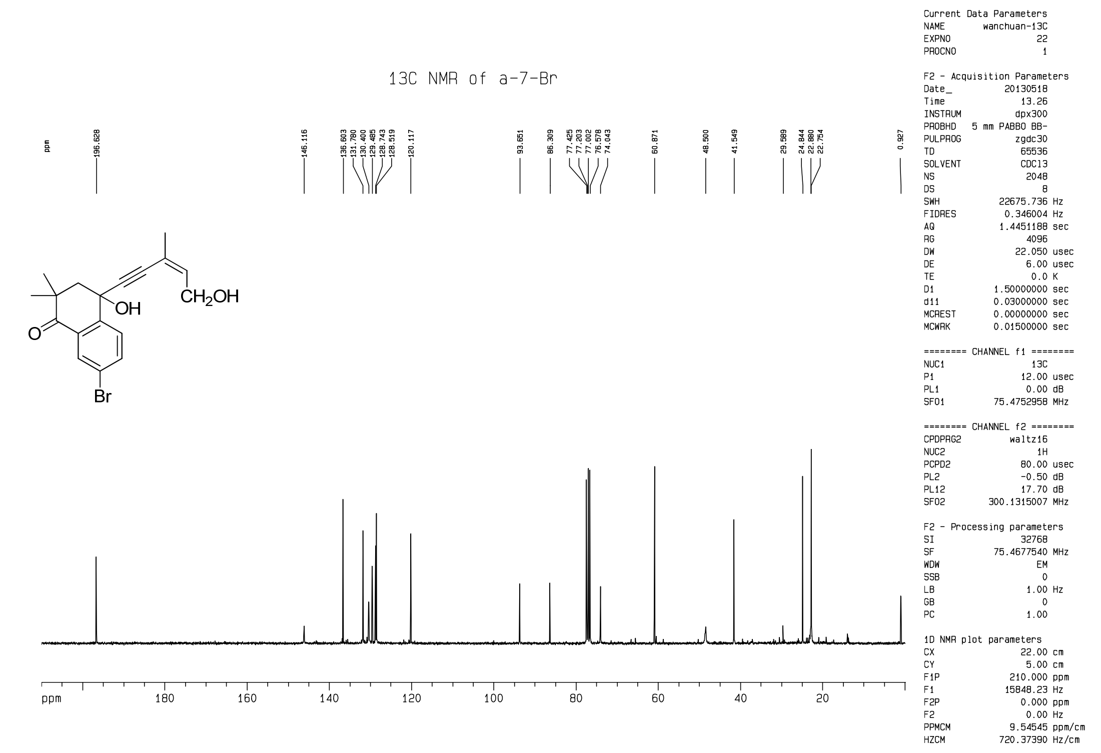

Figure S38. ^{1}H-NMR of compound **6i**.

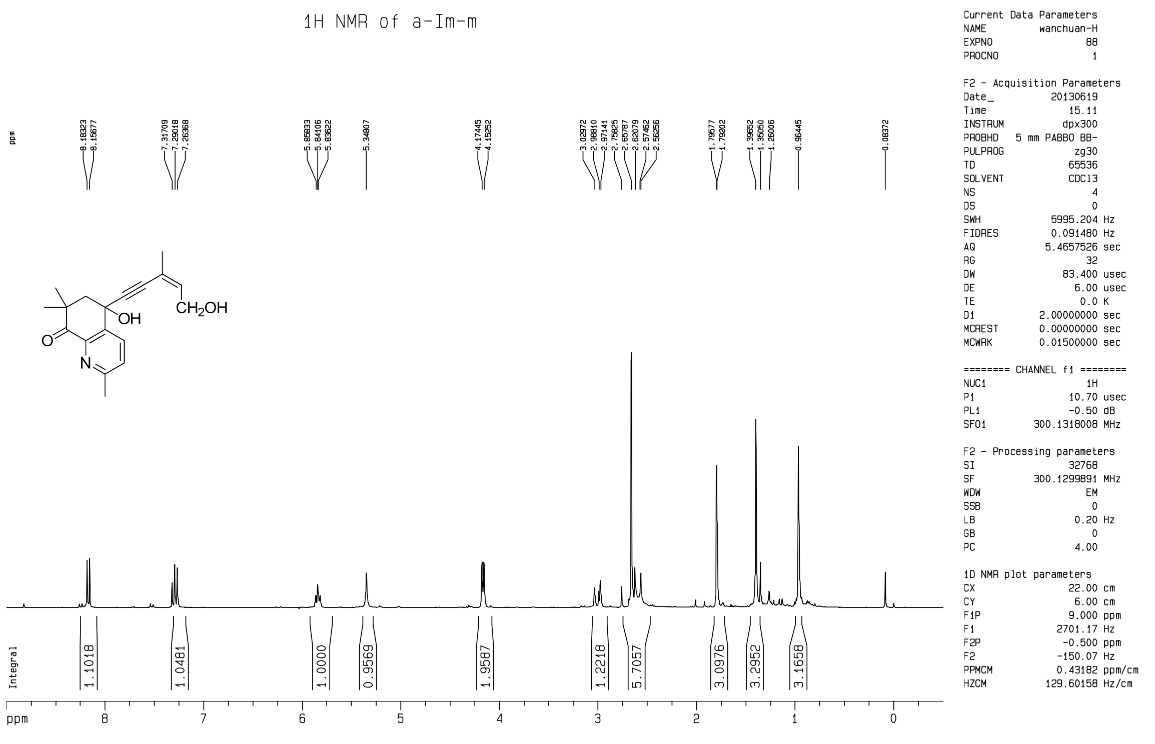

Figure S39. ^{13}C-NMR of compound **6i**.

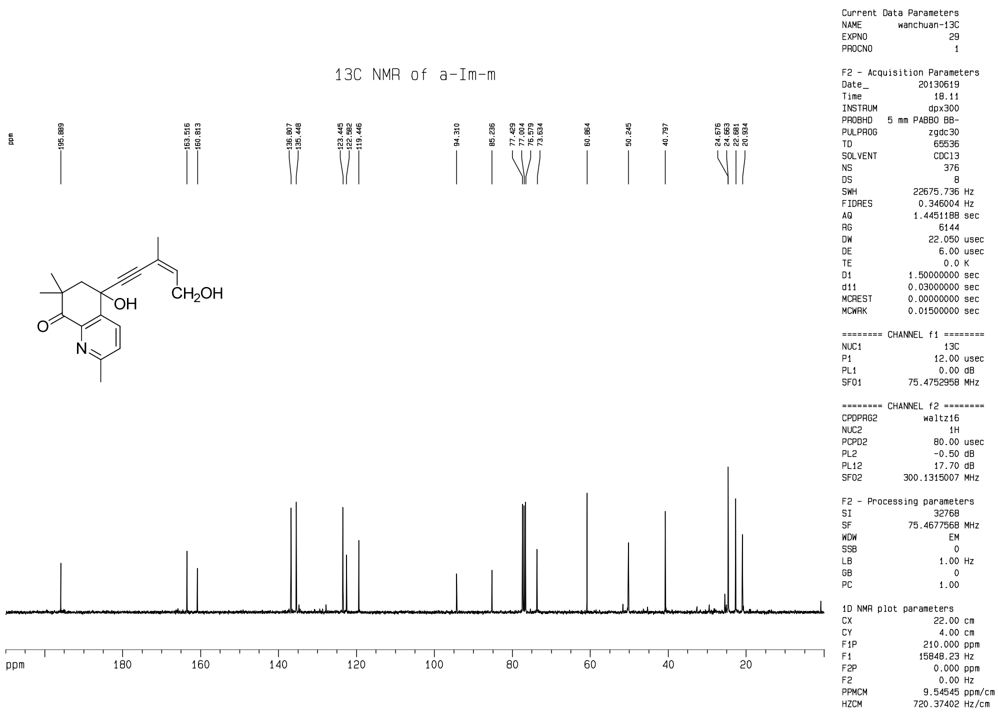

Figure S40. ^{1}H-NMR of compound **6j**.

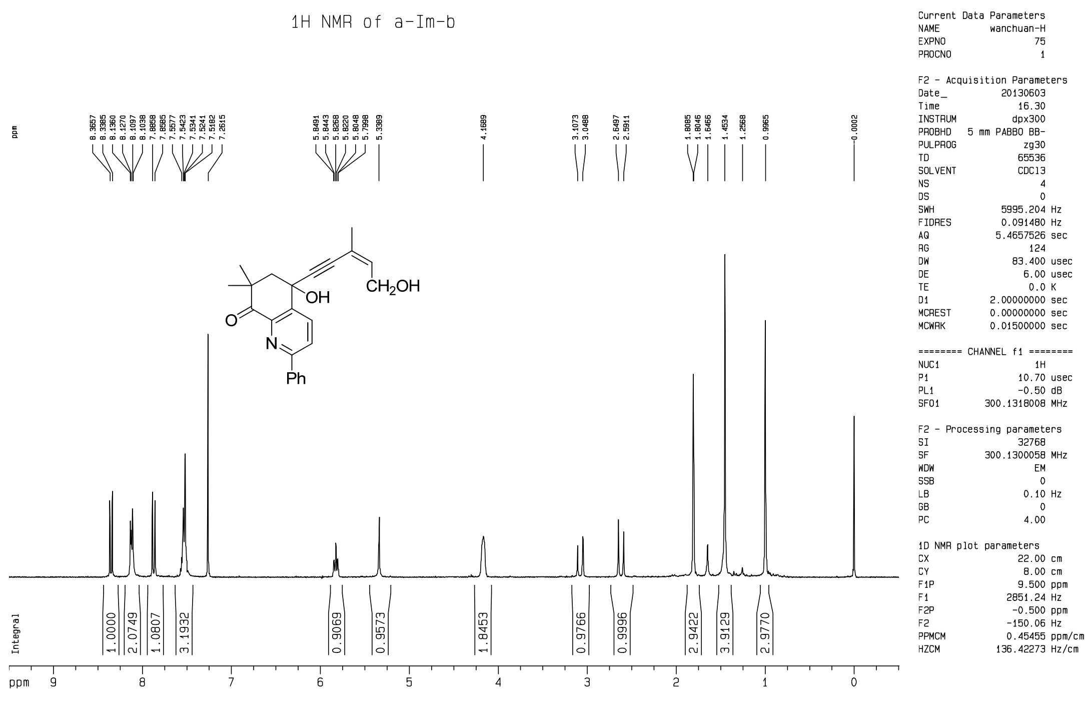

Figure S41. ^{13}C-NMR of compound **6j**.

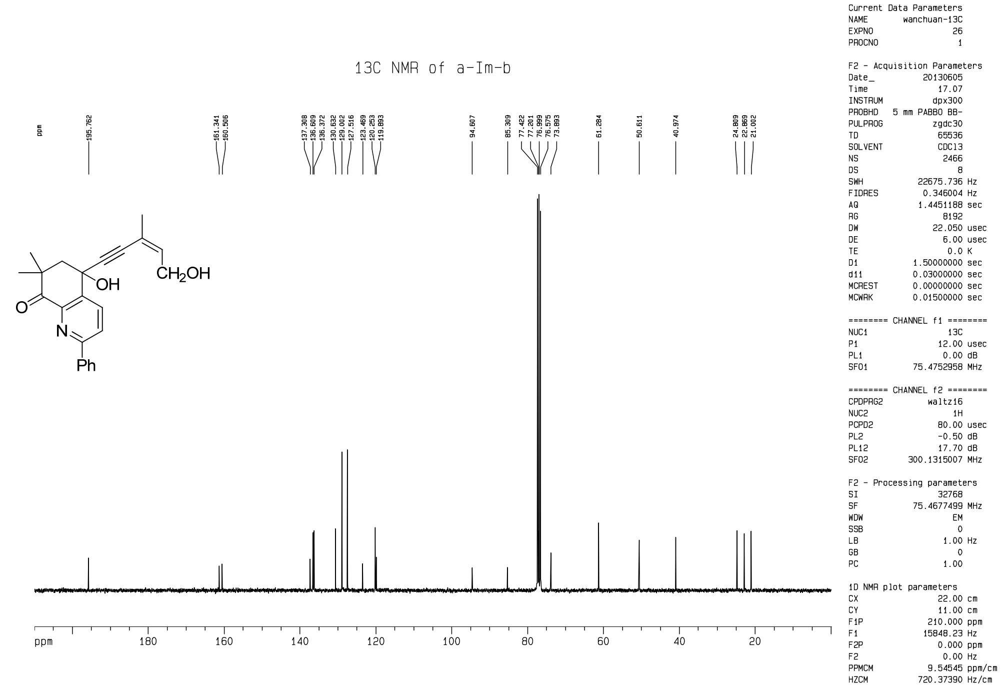

Figure S42. ^{1}H-NMR of compound **6k**.

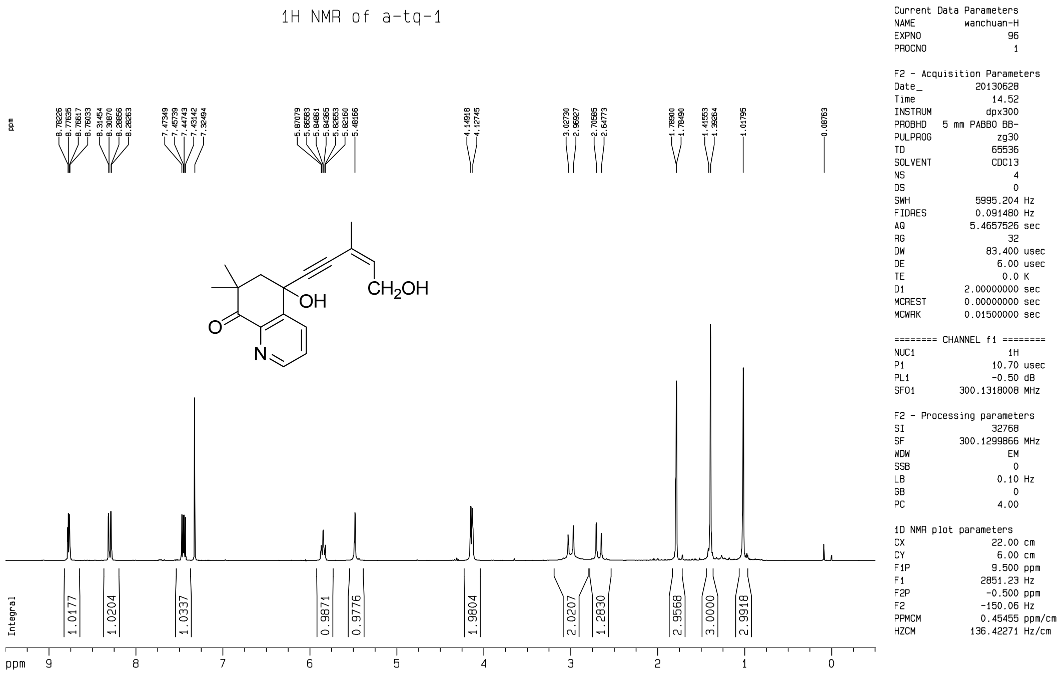

Figure S43. ^{13}C-NMR of compound **6k**.

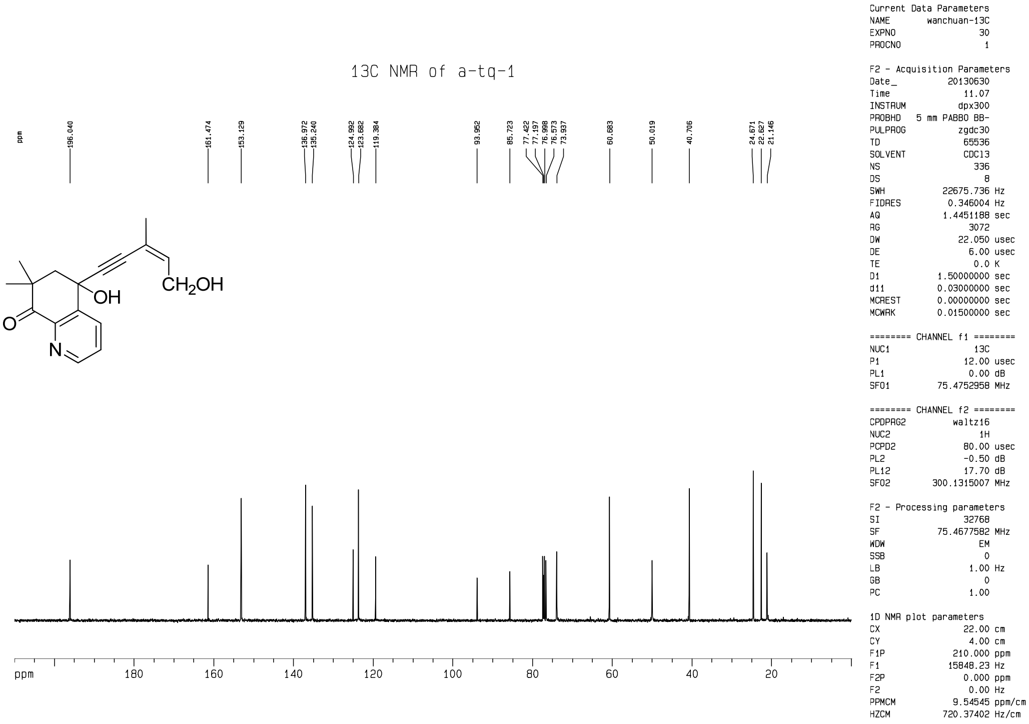

Figure S44. ^{1}H-NMR of compound **7**.

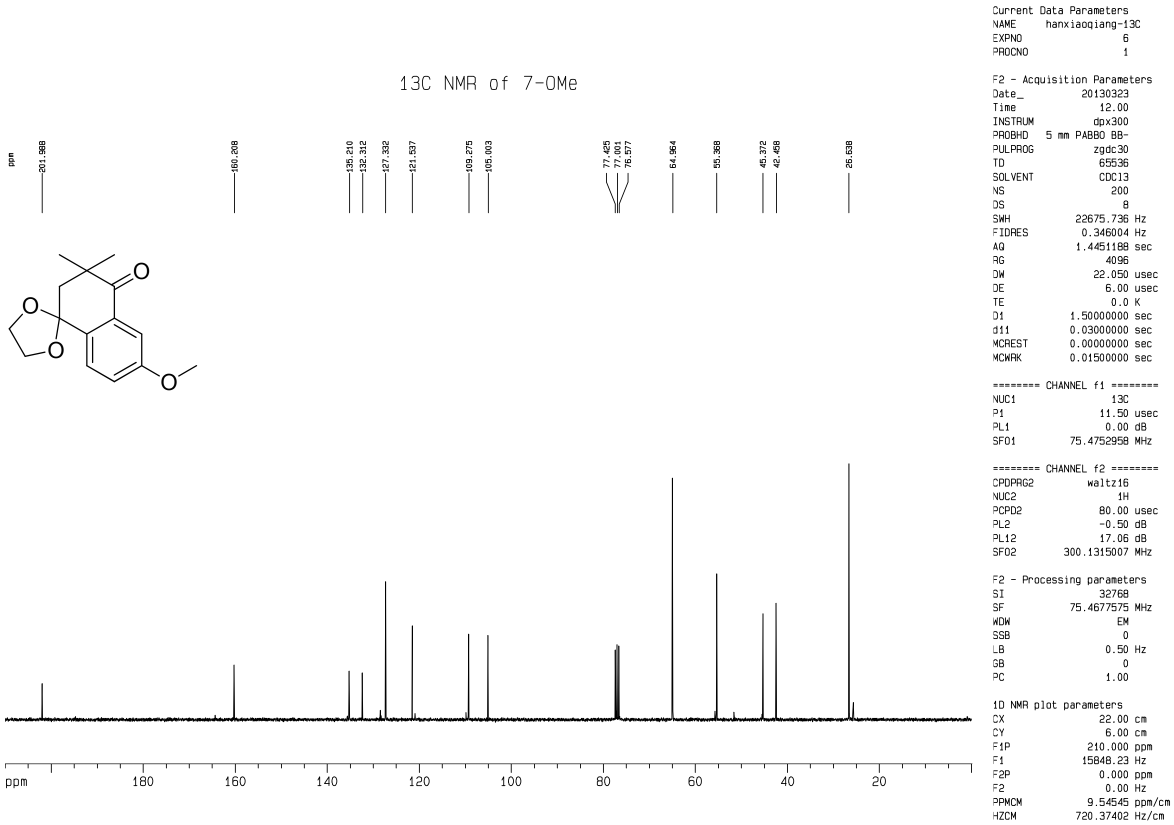

Figure S45. ^{13}C-NMR of compound **7**.

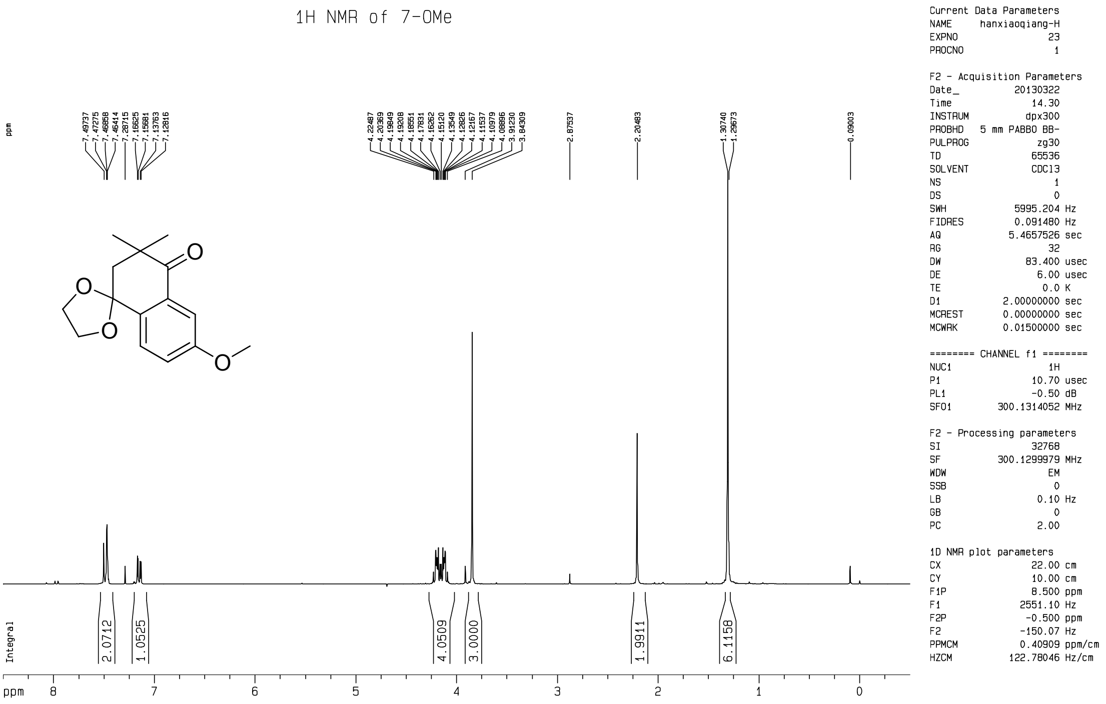

Figure S46. ^{1}H-NMR of compound **8**.

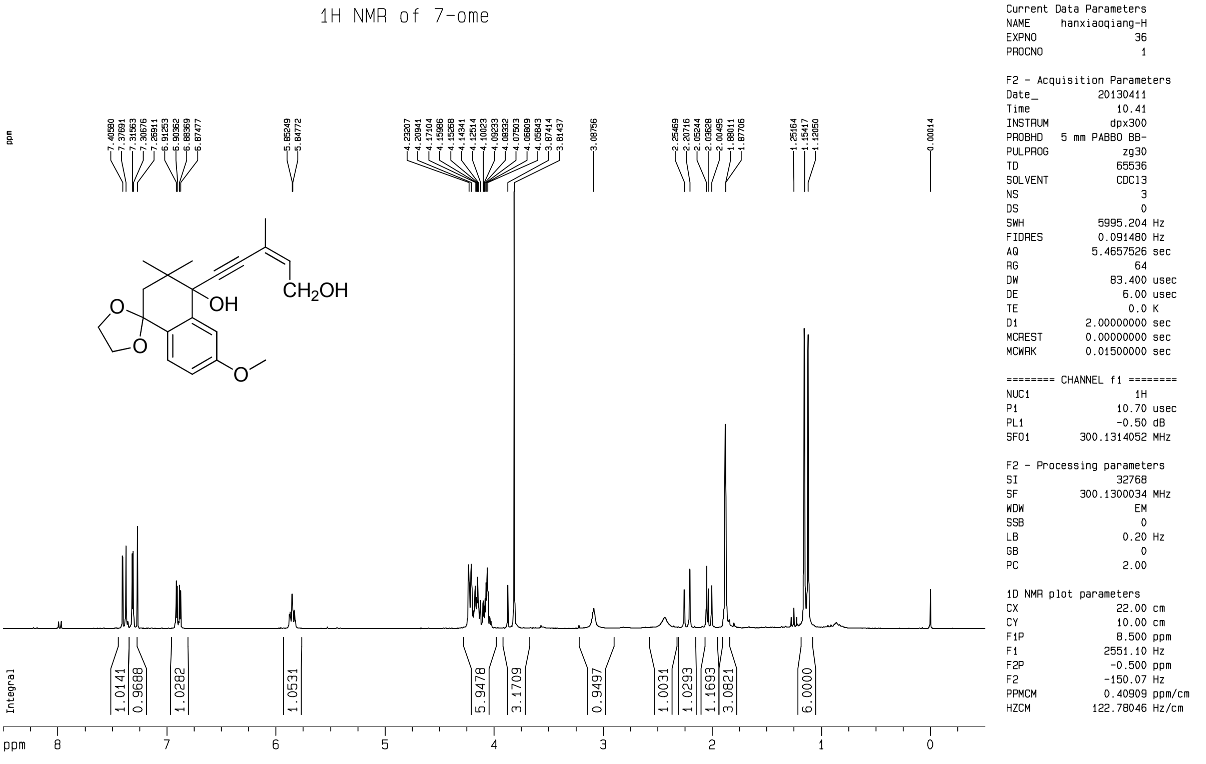

Figure S47. ^{13}C-NMR of compound **8**.

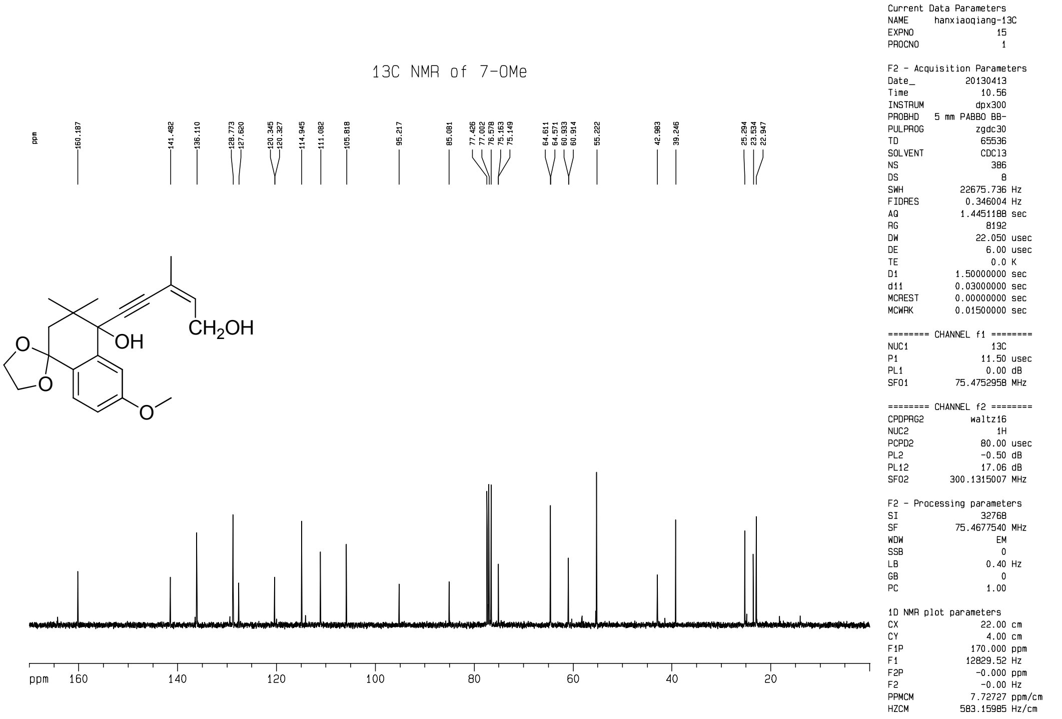

Figure S48. ^{1}H-NMR of compound **5c**.

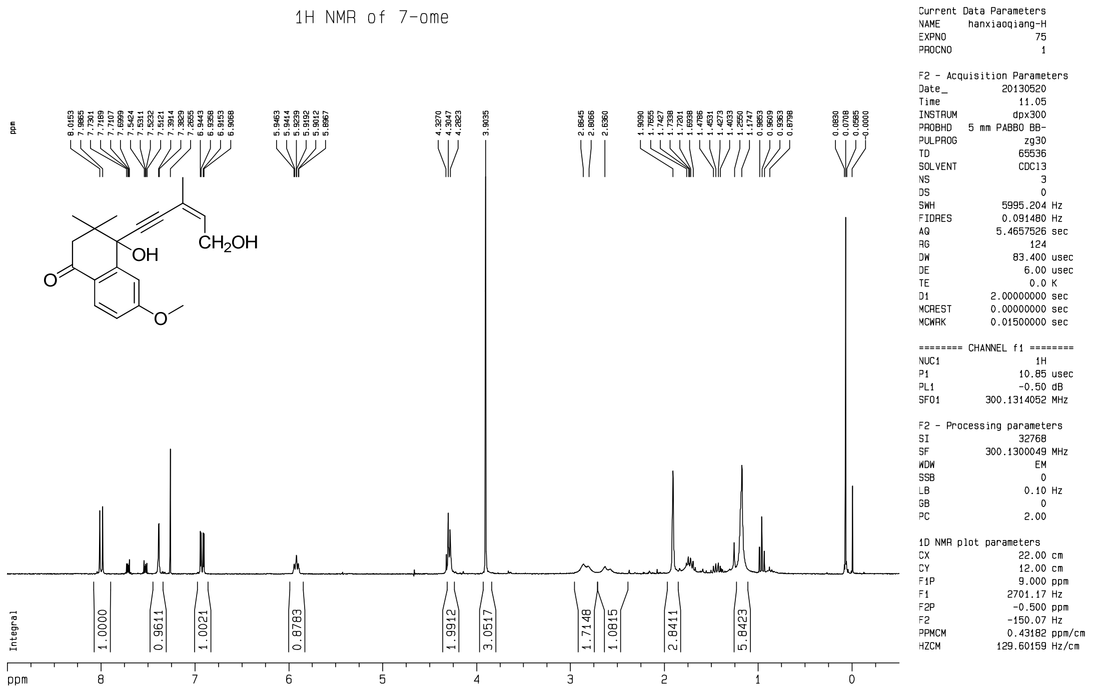

Figure S49. ^{13}C-NMR of compound **5c**.

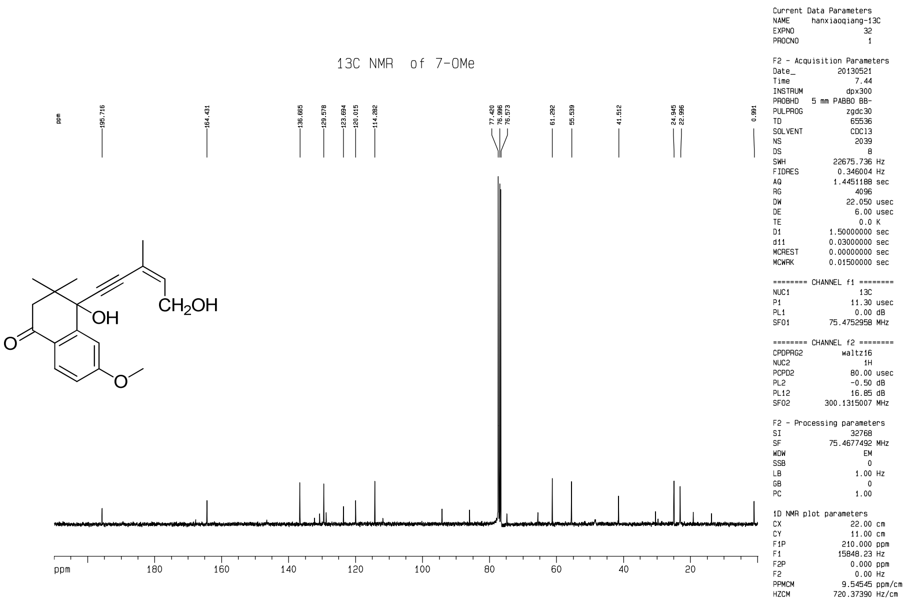

Supplement: Supplementary file 1 [file molecules-18-10776-s001.pdf]
